# Stage-specific dynamic reorganization of genome topology shapes transcriptional neighborhoods in developing human retinal organoids

**DOI:** 10.1016/j.celrep.2023.113543

**Published:** 2023-12-03

**Authors:** Zepeng Qu, Zachary Batz, Nivedita Singh, Claire Marchal, Anand Swaroop

**Affiliations:** 1 Neurobiology, Neurodegeneration, and Repair Laboratory, National Eye Institute, National Institutes of Health, MSC0610, 6 Center Drive, Bethesda, MD 20892, USA; 2 In silichrom Ltd, 15 Digby Road, Newbury RG14 1TS, UK; 3 These authors contributed equally; 4 Lead contact

## Abstract

We have generated a high-resolution Hi-C map of developing human retinal organoids to elucidate spatiotemporal dynamics of genomic architecture and its relationship with gene expression patterns. We demonstrate progressive stage-specific alterations in DNA topology and correlate these changes with transcription of cell-type-restricted gene markers during retinal differentiation. Temporal Hi-C reveals a shift toward A compartment for protein-coding genes and B compartment for non-coding RNAs, displaying high and low expression, respectively. Notably, retina-enriched genes are clustered near lost boundaries of topologically associated domains (TADs), and higher-order assemblages (i.e., TAD cliques) localize in active chromatin regions with binding sites for eye-field transcription factors. These genes gain chromatin contacts at their transcription start site as organoid differentiation proceeds. Our study provides a global view of chromatin architecture dynamics associated with diversification of cell types during retinal development and serves as a foundational resource for in-depth functional investigations of retinal developmental traits.

## In brief

Qu et al. use retinal organoids to measure the interplay between 3D genome organization and gene expression during the differentiation of a complex human neural tissue, showing topology changes at all levels in key retinal genes.

## Graphical Abstract

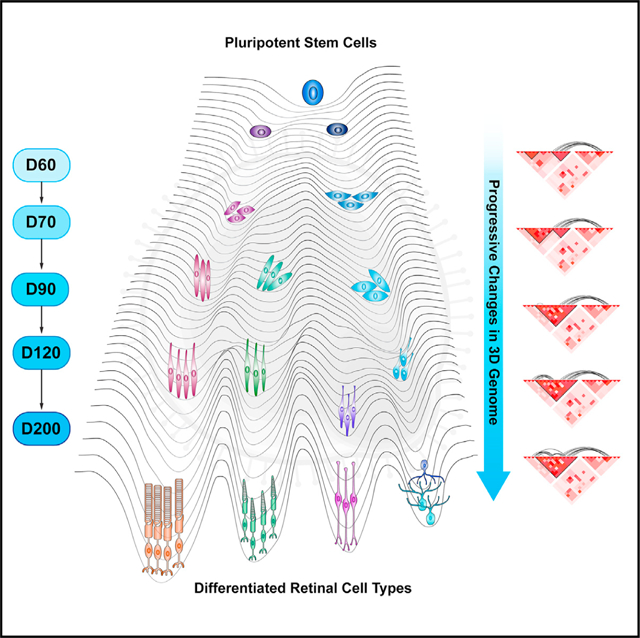

## INTRODUCTION

Organogenesis involves progressive restriction of developmental potential of pluripotent stem cells, resulting in generation of divergent cell lineages with precise spatiotemporal patterns of gene expression. Cell-type-specific decoding of genetic information is extremely precise and orchestrated largely by the inter-relationship of transcription factors (TFs) with the three-dimensional (3D) chromatin organization at specific genomic loci.^1,2^ Higher-order genome topology constrains the activity of *cis*-regulatory elements (CREs) such as enhancers and permits context-dependent multi-valent physical contacts for gene regulation.^3–5^ Controlled chromatin remodeling during development moves relevant CREs in proximity of transcription start sites (TSSs) by chromatin loops to permit TF clustering for guiding expression of specific genes in relevant cell types.^6–9^ Using the genome-wide high-resolution *in situ* chromatin conformation capture (Hi-C) technique, the spatial structure of the 3D genome can be parsed and visualized at multiple levels including megabase-scale A/B compartments, submegabase-scale topologically associated domains (TADs), and kilobase-scale chromatin loops.^10^ High-resolution Hi-C maps can help decipher the hierarchical genome organization associated with establishment and maintenance of the transcriptional landscape, which is required to determine cellular identity and function. Dynamic changes across distinct hierarchical levels of 3D genome architecture are not only a consequence of but also active contributors to decision-making during differentiation and maintenance of functional state.^11–15^

The retina offers a classic paradigm for investigating neural tissue development, with multipotent retinal progenitor cells (RPCs) acquiring competence to differentiate into seven major classes of terminal cells and form a functional laminated structure.^16,17^ Pioneering studies in model organisms have yielded fundamental insights into transcriptional networks that control the development of retinal cells, especially photoreceptors.^18–25^ Remarkable spatiotemporal coordination of differentiation and stratification of retinal cell types are undoubtedly more complex in humans and other primates that include a distinct avascular central region, called fovea, which is designed for high visual acuity.^26,27^ Notably, the fovea and its surrounding macula develop more rapidly compared with the peripheral retina in humans.^28,29^ Transcriptomic, chromatin accessibility as well as epigenomic profiling have begun to uncover the regulatory landscape of human retina and their relation to disease.^30,31^ Genetic mutations that alter TAD structure can lead to ectopic contacts for retina-specific enhancers and drive retinal degeneration.^32^ Incorporation of high-resolution Hi-C data into epigenomic, expression quantitative trait locus (eQTL) and genome-wide association studies (GWASs) has deciphered an integrated chromatin topology map of adult human retina and permitted identification of candidate genes for age-related macular degeneration and glaucoma.^33^

TF-mediated chromatin looping has been proposed to contribute to gene regulation and lineage specification in mouse and human brain development.^34,35^ How multi-scale genome architecture impacts gene expression patterns and consequently differentiation of RPCs to divergent cell types in human retina is still poorly understood. Investigations of human retinal development are complicated by the lack of high-quality tissue samples at relevant stages. Retinal organoids (ROs) derived from embryonic and induced pluripotent stem cells (iPSCs) have emerged as a powerful tool for investigating human retinal differentiation and for disease modeling.^36–39^ These ROs progress through stages that closely recapitulate human fetal retinal development,^40–45^ contain differentiated neurons organized into laminar structures, and even partially mimic retinal function.^46,47^

To comprehensively assess the role of 3D genome architecture in controlling gene expression and directing cell fate decisions, we generated a high-resolution chromatin contact map of developing human ROs by *in situ* Hi-C. We analyzed the topology dynamics across hierarchical levels focusing on the crucial developmental window from RPCs to terminally differentiated retinal cells. Integration of the Hi-C data with corresponding transcriptomic profiles uncovered the relationship between 3D genome reorganization and transcriptional neighborhoods of regulatory loci, including an enrichment of chromatin looping, which in turn facilitates the expression of cell-type-relevant genes. Our study thus highlights the role of genome dynamics in cell fate determination through the regulation of lineage-specific TFs and provides key regulatory insights into human retina development and associated disorders.

## RESULTS

### High-resolution Hi-C reveals fine 3D nuclear dynamics during retinal organoid differentiation

**Figure 1.**
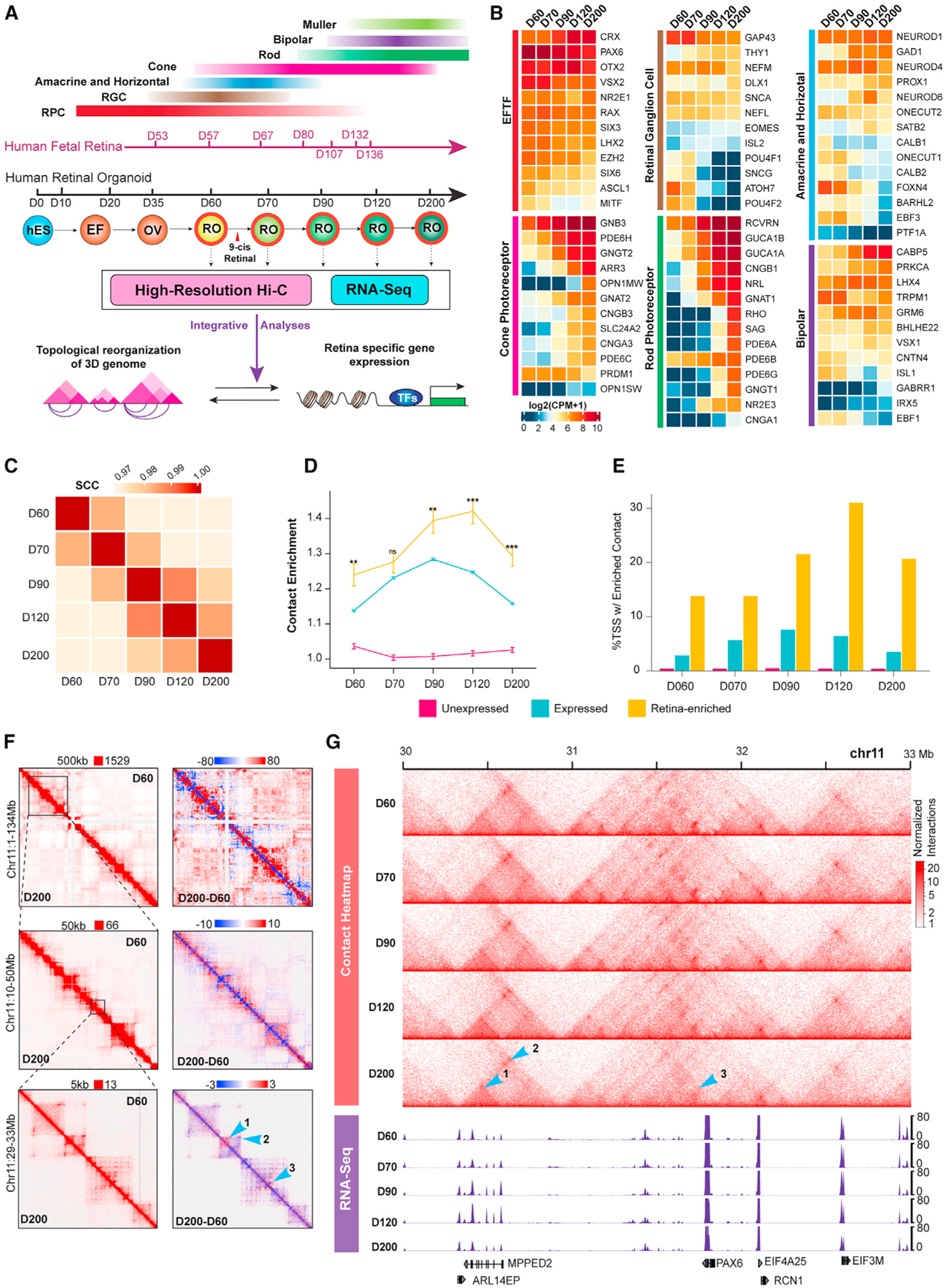
High-resolution chromatin interaction maps uncover changes in genome topology during human RO differentiation (A) Schematic overview of the project design. Retinal organoid differentiation recapitulates the sequential production of cell types across the early stages of human retinal development. Developing human retinal organoids were collected chronologically at five time points indicated by red circles and arrows for Hi-C and RNA-seq analyses. (B) Heatmaps showing gene expression dynamics for retinal cell markers during differentiation. CPM, counts per million. (C) Similarity of contact maps between organoid samples at different time points measured by stratum-adjusted correlation coefficients (SCC). (D) Enrichment ratio of Hi-C contacts at transcription starting sites (TSS) among unexpressed, expressed, and retina-enriched genes during differentiation. Data are represented as mean ± SE. Significance evaluated by permutation test; **p < 0.01 and ***p < 0.001 indicate significant differences (t test) between retina-enriched and other expressed genes at a given time point. (E) Percentage of genes with significantly enriched contacts at the TSS in each category of (D) across differentiation. (F) Hi-C contact maps for organoids at 500 kb (top), 50 kb (middle), and 5 kb (bottom) resolutions. Left column shows Knight-Ruiz balanced maps, with D60 on the upper triangle and D200 on the lower triangle. Right column shows the log_2_ ratio of D200 over D60 for the same region. Blue arrows indicate regions of interest. (G) Top five tracks show contact maps, lower five tracks show gene expression across all time points in CPM. Blue arrows identify regions of interest highlighted in (E). See also Figure S1 and Tables S1 and S2.

We took advantage of an established stable RO differentiation protocol^37,48,49^ and differentiated the H9 human embryonic stem cell (ESC) line up to 200-day-old ROs (Figure S1A) by supplementing the medium with 9-*cis*-retinal at D63 to accelerate photoreceptor maturation.^41^ We confirmed the progression of retinal development by staining for key retinal cell markers (Figure S1B). To elucidate the relationship of genomic architecture and gene expression, we conducted high-resolution *in situ* Hi-C mapping of chromatin contacts and RNA-seq analyses using ROs across five time points (Figure 1A), which model key developmental stages in the human retina.^28,43^ As predicted, expression of eye-field and retinal TFs in ROs is concordant with an increase in most retinal cell-type-specific markers (Figures 1B, S1B, and S1C). However, decreased expression of most ganglion cell marker genes is evident in later stages of RO differentiation, likely because of the loss of ganglion cells (Figures 1B, S1B, and S1C). Together, these data demonstrate progressive retinal neurogenesis in the cultured ROs.

Using *in situ* Hi-C, we sequenced 4.283 billion total read pairs across five time points (832–908 million read pairs per time point, Table S1). The sequencing libraries contained >90% valid interactions (range: 90.0%–94.1%) and a consistently low proportion of *trans*-interactions (range: 15.2%–26.2%), indicating a high-quality dataset. From the sequencing, we obtained 2.789 billion total valid chromatin contacts (range: 519–604 million valid contacts per time point) and achieved ~5 kb resolution (range: 4.76–5.20 kb) for each of the first four time points (D60–D120) and 9.79 kb resolution for D200. We then performed hierarchical clustering of the stratum-adjusted correlation coefficient (SCC) averaged across chromosomes, as previously described.^50^ The first two samples (D60 and D70) and the last three samples (D90 through D200) exhibit high similarity (Figure 1C), indicating greater changes in genome structure from D70 to D90 compared with other time points. Similarly, chromatin contacts are increased at the TSS of retina-enriched genes at D90 (Figure 1D; Table S2). Moreover, the proportion of retina-enriched genes significantly enriched for contacts increases at D90 and exceeds the proportion observed across expressed non-retina-enriched genes or non-expressed genes (Figure 1E; Table S2).

Our data demonstrate that the genome organization is transformed in a unidirectional manner during retinal organoid differentiation: the chromosomal contact profile shifts progressively from the conventional proximal contact-enriched state to include increased long-range distal interactions (Figure S1D). The genome-wide interactions can be explored at multiple hierarchical levels, including chromosomal compartments, TADs, and loops, and at different resolutions. As an example, we show changes in chromosome 11 genome structure observed at different resolutions (Figure 1F); at high resolution, fewer interactions are evident at the *PAX6* TSS, coincident with lower expression at later stages (Figure 1G). In contrast, increased contact frequency at *MPPED2*, potentially linked to neuronal development, is accompanied by higher mRNA expression (Figure 1G). Thus, our high-resolution Hi-C data capture the interactions of genes related to human retina differentiation in organoid culture.

### Compartment changes are associated with gene type and function

**Figure 2.**
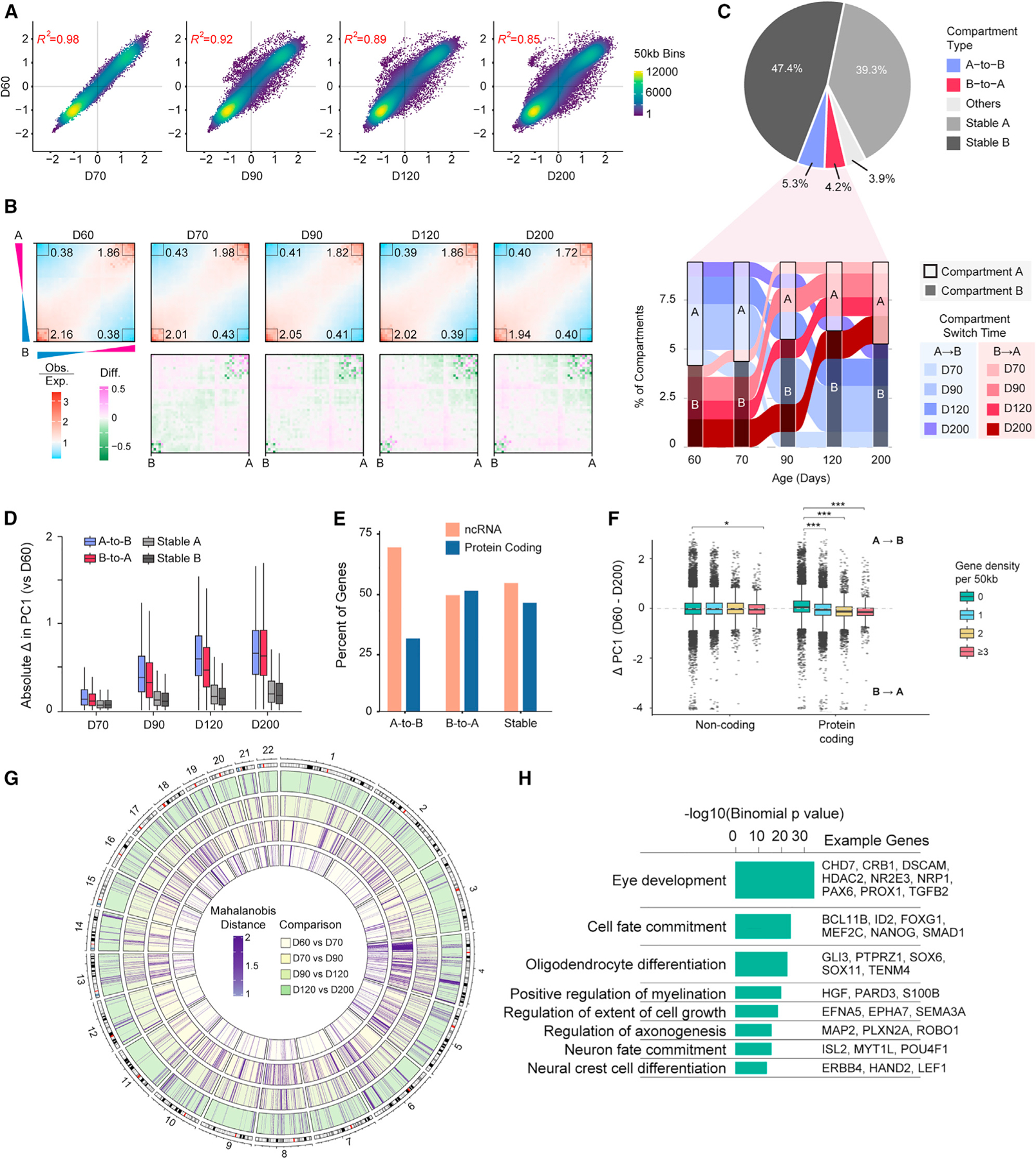
Compartment dynamics in developing human ROs (A) Correlation between compartment score at D60 and compartment scores in all other time points. Each point represents one 50 kb genomic region. Density in points is indicated by the color code. Correlation between each pair of samples is indicated on the corresponding plot (Pearson’s *R*^2^). (B) Frequency of inter- and intra-compartment interactions at each time point (top) and change in interaction frequency relative to D60 (bottom). Printed numbers in upper plots capture relative enrichment of interaction types (top right: A-A interactions; bottom left: B-B interactions; top left and bottom right: A-B or B-A interactions). (C) Proportion of 50 kb genomic bins with stable or dynamic compartments throughout differentiation (top). Proportion of stable compartment switches occurring by time point during differentiation (bottom). (D) Absolute changes in compartment score (PC1) relative to D60 for each stable compartment type identified in (C). Boxplots show the median and interquartile range (IQR); whiskers mark 1.5x the IQR. (E) Proportion of non-coding RNA (ncRNA) vs. protein-coding genes overlapping genomic regions with stable or switching compartments. (F) Change in compartment score from D60 to D200 split by gene type and density. Boxplots represent the median and interquartile range (IQR); whiskers mark 1.5x the IQR; data beyond 1.5x the IQR are plotted as individual points. *p < 0.05 and ***p < 0.001 indicate significance evaluated by t test. (G) Change in compartment score between consecutive time points (darker purple = greater difference). Outermost track represents each autosome, and the inside four tracks indicate the Mahalanobis distance for each sample compared with D60. Only loci with Mahalanobis distance of >1 are shown. (H) Enriched GO terms among genes in regions with compartment score changing from D90 to D120 (Mahalanobis distance >1); p value was calculated by a two-sided binomial test. A selection of genes associated with each term are presented; full results for all time points are provided in Table S3. See also Figure S2 and Table S3.

Spatial segregation of chromatin in open euchromatin (i.e., A compartment, associated with active genes) and closed heterochromatin (i.e., B compartment, associated with inactive genes) regions is a hallmark of genome organization.^51^ We segmented chromosomes into A and B compartments based on principal-component analysis (PCA) at 50-kb resolution and observe a progressive genome-wide decrease in the compartment score (PC1) correlation between D60 and other time points during organoid differentiation (Figure 2A). The largest shift in PC1 occurs between D70 and D90 (Figure 2A), and the percentage of the genome residing in A compartment decreases steadily from D70 to D120 with a slight increase by D200 (Figure S2A). To assess the degree of spatial separation of compartments,^52^ we measured the overall interaction strengths within and between A and B compartments and visualized these differences using saddle plots. Although we observe the largest shift in contact patterns and compartmentalization between D70 and D90, the largest increase in A-A contacts and decrease in B-B contacts occurs between D60 and D70 (Figure 2B). Thus, second-order inter-compartment interaction changes appear to precede other modifications in primary chromatin structure.

To quantify changes from euchromatin to heterochromatin during differentiation, we counted loci that stably switched between the A and B compartments. Consistent with previous studies on cell differentiation,^53,54^ the vast majority of the genome resides in stable compartments across all five differentiation time points. Among the 13.3% compartments that change during differentiation, we observe more genomic loci undergo stable A-to-B switches (145.8 Mb) compared with stable B-to-A switches (83.9 Mb) (Figure 2C; Figure S2B). Enrichment of A-to-B switching is also observed during differentiation of neurons and cardiomyocytes from pluripotent stem cells.^34,55^ For genomic regions undergoing stable compartment switches, the largest change in compartment score (PC1 value) occurs between D70 and D90 (Figure 2D). The distribution of compartment switching is not uniform across chromosomes, and we note a particular concentration on the p arm of chromosome 4 (Figure S2C). Furthermore, non-coding RNAs are enriched in regions undergoing A-to-B compartment switches, whereas B-to-A compartment switching regions and stable regions contain a relatively equal proportion of coding and non-coding genes (Figure 2E). We note that regions with a higher density of protein-coding genes tend to shift toward A compartment throughout development, whereas gene poor regions move toward B compartment; notably, this pattern is largely absent in non-coding genes (Figure 2F). Additionally, individual 50 kb windows with the highest density of protein-coding genes tend to shift toward A compartment, whereas regions with the highest density of non-coding genes shift toward B compartment (Figure S2D). Thus, preferential reorganization of non-coding sequences into heterochromatic regions offers one possible conduit for maintenance of the differentiated state.

We then employed dcHiC^56^ to calculate the Mahalanobis distance (MD) between time points to identify changes in compartment score that may not culminate in a compartment switch. By this measure, we detect widespread shifts in compartments across the genome (Figure 2G; Table S3; Figure S2E) and again note a clustering of changes on chromosome 4p, especially in a region of very low gene density from 25 to 40 Mb (Figure S2F). We interrogated ontology of genes located in genomic regions with changes in compartment at each time interval using GREAT.^57^ Although the largest shifts in compartment score are identified between D70 and D90, the genes residing in these regions are primarily related to kidney development and move toward B compartment. In contrast, the regions shifting compartment score between D90 and D120 were enriched for GO terms associated with eye development and cell fate commitment (Figure 2H). Thus, compartment changes occurring from D70 to D90 seem to prime the genome topology for subsequent changes in expression of genes (from D90 to D120) involved in differentiation of late-born retinal cell types.

### Compartment switching is associated with changes in expression of retinal genes

**Figure 3.**
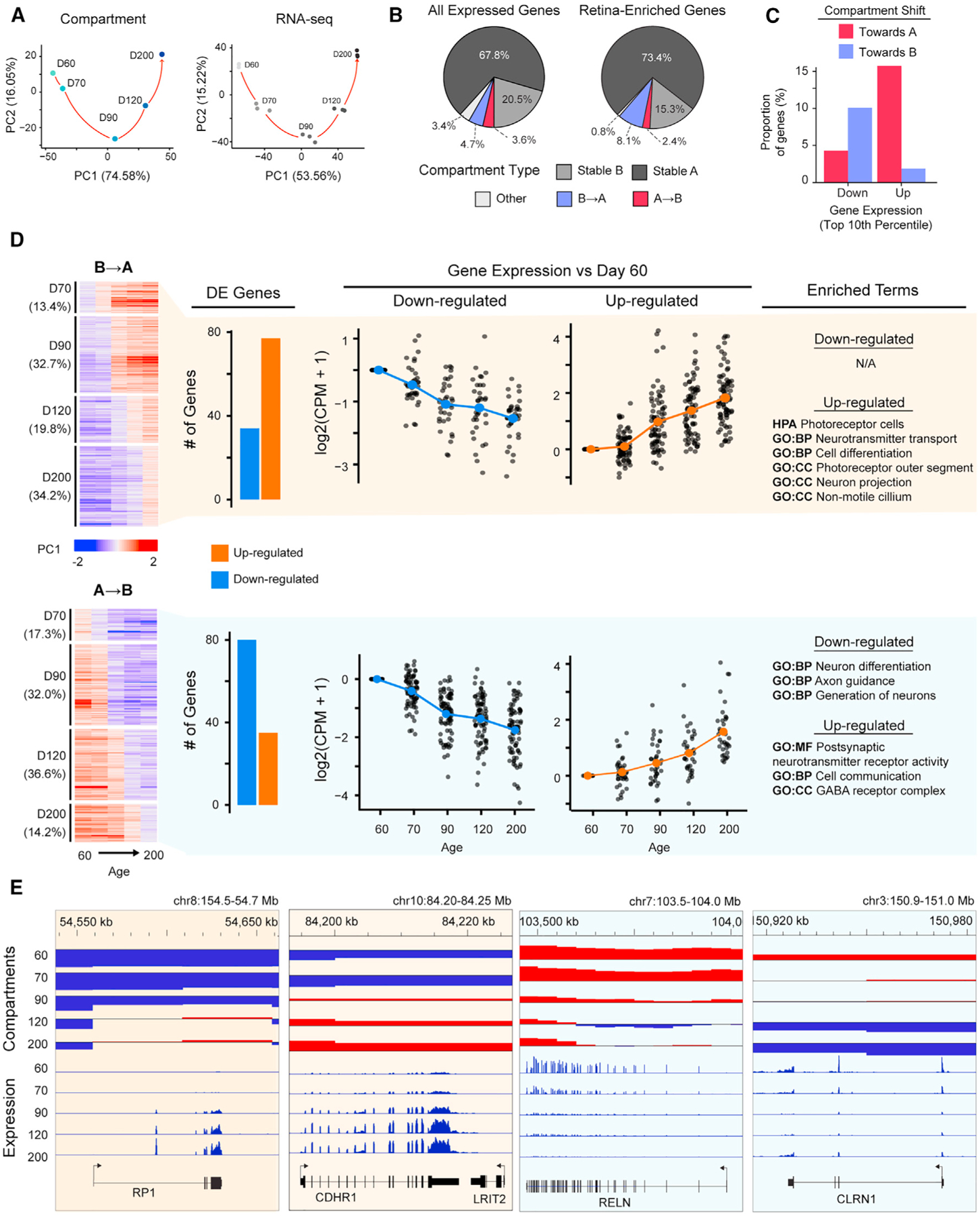
A/B compartment switches partially correlate with gene expression changes during RO differentiation (A) PCA plots of Hi-C compartment score at 50 kb resolution (left) and normalized RNA-seq read counts (right) during differentiation. (B) Proportion of all expressed genes (CPM >1, n = 25,946; left) and retinal-enriched genes (Table S2, n = 134; right) across each compartment type. (C) Proportion of genes with largest change in expression (top 10% upregulated and top 10% downregulated, see STAR Methods) found within stably increasing or decreasing compartments. (D) PC1 values for all stably switching compartments, with the proportion of compartments switching per time point listed alongside (left). Proportion of differentially expressed (DE) upregulated and downregulated genes overlapping with stably switched compartments (center). Expression change in DE genes compared with D60 with top enriched ontology terms for each group identified (right). (E) Examples of differentially expressed genes residing in compartments that switch during differentiation. Upper tracks show compartment score; lower tracks show expression in CPM. See also Figure S3 and Table S2.

We visualized the correlation between genome topology and gene expression as PCA plots and noted a highly similar pattern of chronological change in compartment scores and transcriptomes for ROs during differentiation (Figure 3A). Notably, most of the expressed (67.8% out of 25,946) as well as retina-enriched (73.4% out of 134, Table S2) genes reside in stable A compartments (Figure 3B); however, 9.5% of retina-enriched genes switch from B to A compartment during development. The proportion of retina-enriched genes switching from B to A is higher than the proportion observed across all expressed genes (Figure 3B) and significantly more than expected based on permutation testing (mean: 4.41%; SD: 1.90%; Figure S3A). Given the enrichment of retinal genes in A compartment and in B-to-A switches, we hypothesize that compartment conversions, at least in part, coordinate large-scale activation or repression of retinal lineage-specific genes during RO differentiation.

The top 10% of the genes (n = 114) showing a stable increase in expression during differentiation are more frequently detected in compartments shifting toward A rather than toward B, whereas genes having the strongest stable downward expression (n = 119) are present more frequently in compartments shifting toward B (Figure 3C). Accordingly, compartments that switch from B to A are enriched for upregulated differentially expressed (DE) genes, whereas those shifting from A to B include more downregulated DE genes (Figure 3D). Upregulated genes in B-to-A compartments show enrichment of terms associated with photoreceptor and neurotransmitter function, such as *RP1* (Figures 3D and 3E). In contrast, downregulated genes in A-to-B compartments are related to broader neuron development (e.g., *RELN*) (Figures 3D and 3E).

### Altered internal and external interactions in TADs affect retinal gene expression

We then partitioned the genome into TADs using DomainCaller at 10 kb resolution^58^ and noted that the size distribution of TADs is constant throughout differentiation (Figure S4A). We then divided TADs into active TADs (TADs located in A compartment, termed as A-TADs) and repressive TADs (TADs located in B compartment, termed as B-TADs). Consistent with studies in other species and/or cells,^33,59–61^ we note that B-TADs are larger than A-TADs (Figure S4A). Thus, B-TADs occupy a larger proportion of the genome though the total number of B-TADs is less than the number of A-TADs at each time point (Figure S4B). Furthermore, the overall proportion of TADs in compartments is relatively stable across development.

**Figure 4.**
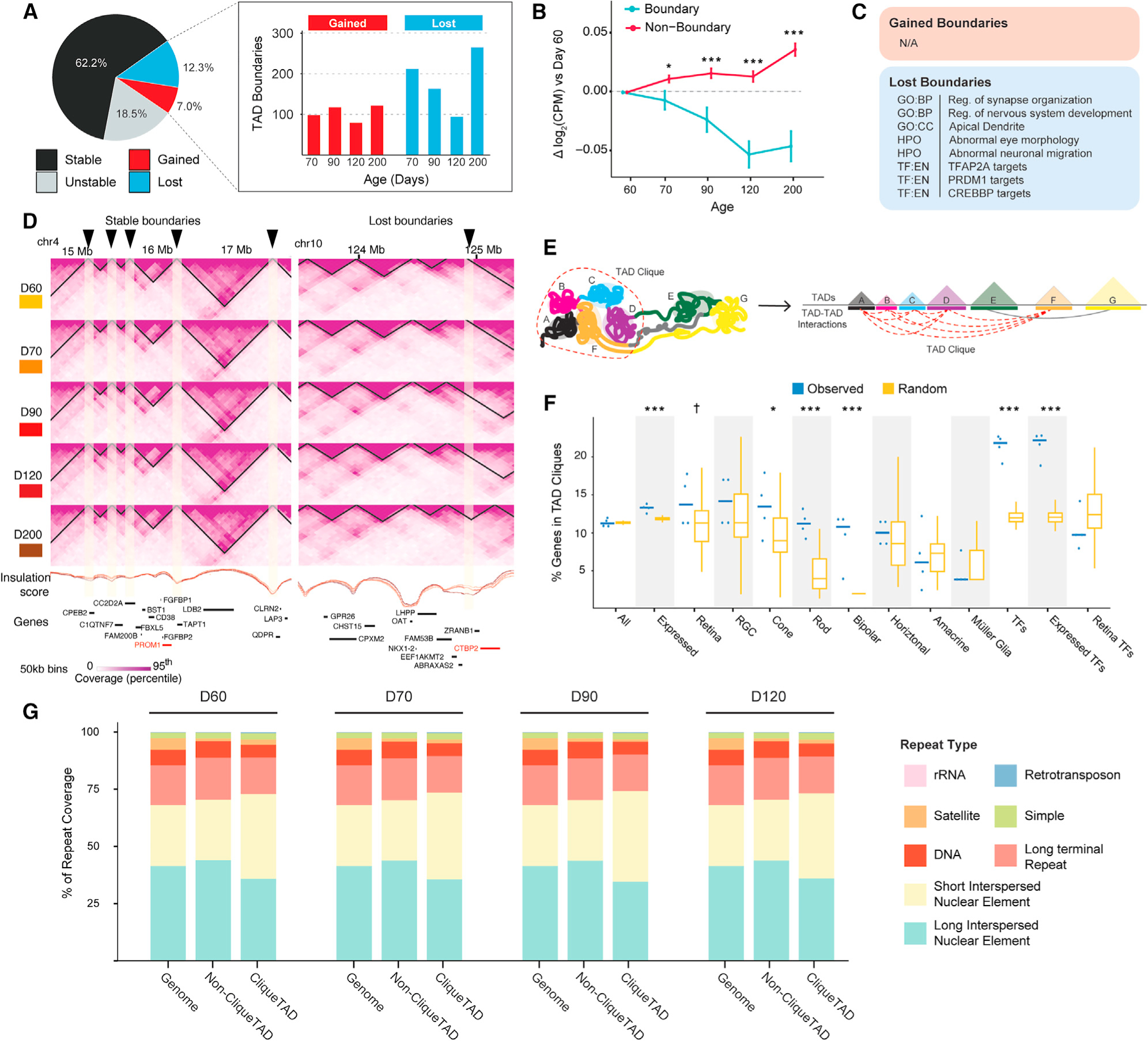
Dynamic changes of TAD structure and corresponding gene expression during human RO differentiation (A) Proportion of stable and dynamic TAD boundaries during differentiation (left). The number of TAD boundaries stably gained or lost at each time point (right). (B) Expression changes for genes with a TSS near a TAD boundary (±10 kb) compared with those in non-boundary regions. Data are represented as mean ± SE. Significance evaluated by t test; *p < 0.05 and ***p < 0.001 indicate significant differences between boundary and non-boundary genes at a given time point. (C) GO terms enriched among expressed genes near (±10 kb) gained and lost boundaries. (D) Example genomic interactions at stable (left) and lost (right) TAD boundaries during RO differentiation. The arrowheads on top indicate the locations of the gained (left) or lost (right) TAD boundaries. Insulation score in each differentiation time point is shown as a line plot and colored according to their name indicated in the legend. Select gene locations shown at bottom with genes of retinal interest highlighted in red. (E) Schematic illustrating the concept of TAD cliques, i.e., clusters of TADs with significant interactions between themselves. (F) Percentage of various gene types with TSS residing in a TAD clique. Each blue point represents one observed time point; horizontal line represents the median of each group. Yellow boxplots represent the null expectation obtained from 100 randomly shuffled clique datasets with the median and interquartile range (IQR); whiskers mark 1.5x the IQR. Differences between real and random data were tested via Wilcoxon rank-sum test, †p < 0.1, *p < 0.05, and ***p < 0.001 indicated above comparisons. (G) Distribution of repeat classes across the entire genome, within TADs not forming cliques and within TADs forming cliques. See also Figure S4 and Table S2.

Initially, the segmentation of the genome into TADs was considered to be relatively conserved and stable across cell types,^11,58^ but later studies have revealed a trend of decreasing TAD numbers during somatic cell differentiation.^34,55^ Similar to these later studies, we observe a 10% reduction in the number of TADs (from 3,967 to 3,557) during RO differentiation from D60 to D200. We then interrogated the TAD boundary changes throughout RO differentiation. Overall, 38% of TAD boundaries are changed during differentiation, including 7% that are stably gained and 12.3% stably lost (Figure 4A). TAD boundaries have a significant effect on expression, as genes residing near TAD boundaries show reduced expression compared with genes with TSS outside TAD boundary regions (Figure 4B). Moreover, this difference in expression progressively increases during differentiation. A similar number of TAD boundaries are gained between each time point examined, whereas TAD boundaries are largely lost at D70 and D200 (Figure 4A). To assess whether dynamic TAD boundaries might correlate to developmental progression, we performed gProfiler^62^ enrichment analysis on genes located near (±10 kb) gained and lost boundaries. Whereas no terms are enriched near gained boundaries, the genes near lost boundaries show enhanced terms related to synapse organization, neuronal migration, eye development, and targets for TFs linked to retinal cell-type development (Figure 4C). For example, *PROM1*, an important gene associated macular disease in human retina,^63^ is located in nearby a stable TAD boundary. Another key gene for retinal development, *CtBP2*, which has been proved to be essential for GABAergic amacrine cells development^64^ and involved in the protect RGC from apoptosis in the retina^65^ is located nearby a lost TAD boundary (Figure 4D).

Stable TADs may further contribute to lineage specification via unique contact patterns within and across TADs.^11,66^ To explore the relationship between stable TADs and retinal gene expression during RO differentiation, we clustered stable TADs by domain score, i.e., the ratio of intra-TAD contacts to inter-TAD contacts^67^ (Figure S4C). Among the nine clusters, we identified two (clusters 4 and 9) with TADs enriched for genes associated with retinal function and development (Figures S4C and S4D). In both clusters, domain score is reduced at D90 indicating more frequent contact within TADs containing these genes rather than across TADs. Conversely, we detect one low domain score cluster (cluster 8) that includes genes associated with neuron development (Figure S4E). Together, these data suggest that lineage-specific genes may be concentrated in TADs with their regulatory elements limiting the need for inter-TAD contacts.

Long-range inter-TAD agglomerations (i.e., TAD cliques) are proposed to be associated with many key features of chromatin organization^68^ and play an important role in development.^12,69^ To further explore the relationship between TADs and retinal development, we called TAD cliques as previously described^12^ (see STAR Methods, Figure 4E). TAD cliques are enriched in the A compartment (Figure S4F). Furthermore, genes residing within TAD cliques tend to have higher expression than those in non-clique TADs (Figure S4G) and TAD cliques are particularly enriched for TFs and marker genes associated with rods, cones, and bipolar cells (Figure 4F; Table S2). For example, the rod bipolar cell signal transduction gene *GRM6* is located in a region with a growing TAD clique, whereas the hippocampal neuron axon formation related kinesin protein *KIF13B* resides in the region of a shrinking TAD clique. *CASZ1*, a rod photoreceptor zinc finger TF gene, is located in the TAD clique-maintained region (Figure S4H). Curiously, long-interspersed elements (LINEs) tend to be enriched in TAD cliques with a corresponding depletion of the short-interspersed elements (SINEs) across multiple cell lines^68^; yet we identify the opposite in the developing ROs (Figure 4G). LINEs and SINEs are implicated in altering chromatin structures, affecting transcription, and even regulating embryonic development^70,71^; therefore, TAD cliques may help regulate retinal development by interacting with these repetitive elements. Thus, the 3D genome topology of developing ROs is dynamically rewired at the TAD level, and even TADs with stable boundaries undergo changes in inter- and intra-TAD contact frequencies and in dynamic higher-order multi-TAD cliques.

### Interactions are stably rewired at genes involved in developing human ROs

**Figure 5.**
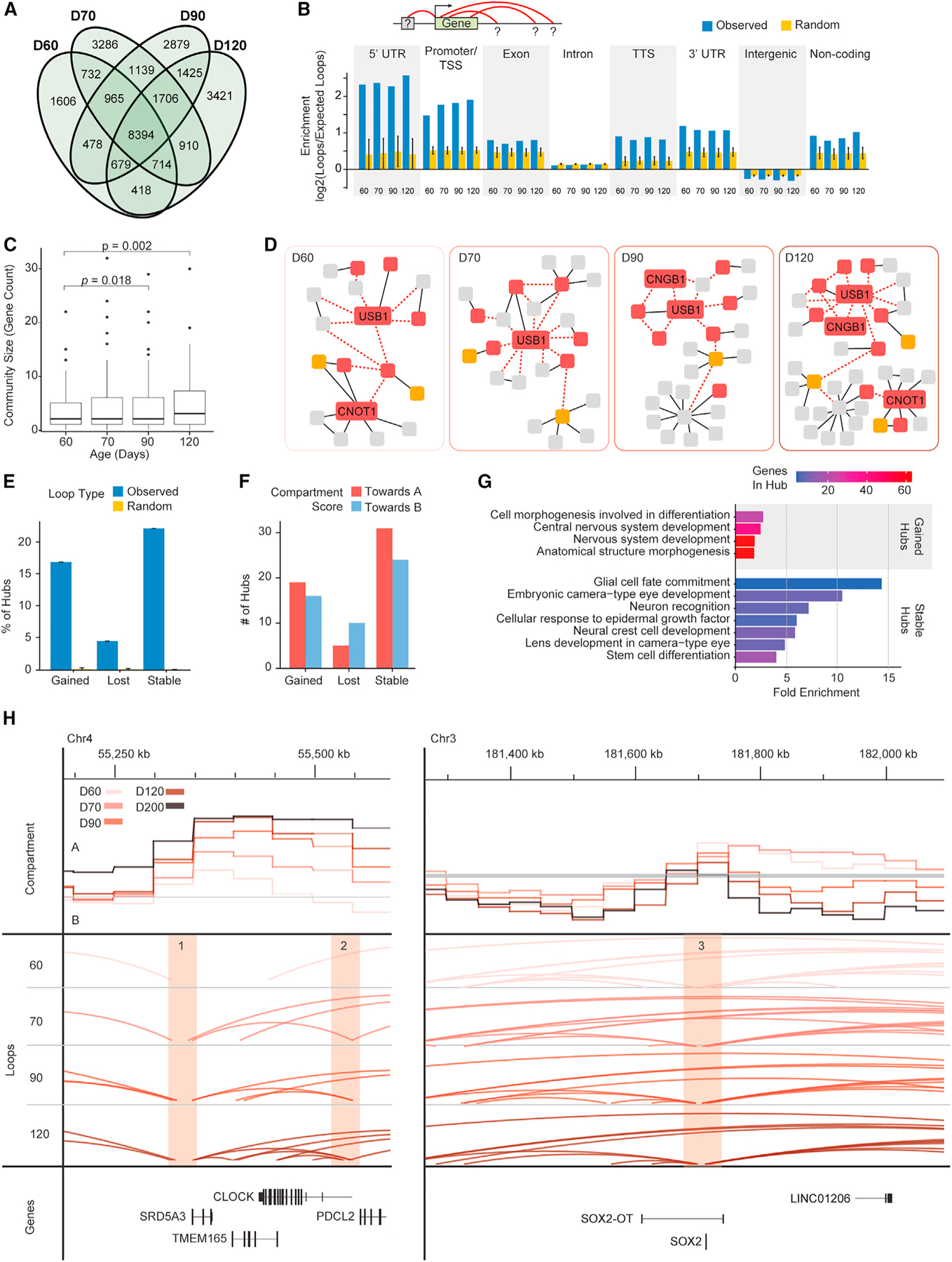
Regulatory rewiring mediated by dynamic chromatin loops and loop hubs (A) Number of shared and unique loops for each time point D60 through D120. (B) Enrichment of observed loop contacts from gene body to various genomic features compared with randomly shuffled loops. The blue columns indicate the observed enrichment; the yellow columns represent the mean ± SD of enrichment detected in 100 randomly shuffled loop sets. (C) Changes in gene community size during differentiation for community containing retina-enriched genes. Boxplots represent the median and interquartile range (IQR); whiskers mark 1.5x the IQR; data beyond 1.5x the IQR are plotted as individual points. p values indicate significance evaluated by t test. (D) An example of gene community changes over differentiation. Boxes indicate gene loci. Gray boxes are genes expressed at <1 CPM; orange boxes are genes expressed at 1–10 CPM, and red boxes are genes expressed at >10 CPM. Black solid lines indicate direct overlap between genes; red dotted lines indicate genes interacting via chromatin loop. Select gene names included, full communities with all gene names submitted to GEO accession numbers GSM7173198-GSM7173201, GSM7173198, GSM7173199, GSM7173200, and GSM7173201. (E) Percent of observed chromatin loop hubs present at >1 time point and that are stably gained, lost, or stable during differentiation compared with loop hubs obtained from randomly shuffled loops. Error bars on random data indicate the mean ± SD of 100 randomly shuffled loop sets. (F) Number of dynamic and stable loop hubs progressively shifting toward A or B compartments during differentiation. (G) Selected GO terms enriched among genes overlapping gained and stable loop hubs during differentiation. No terms are enriched among genes overlapping lost loop hubs. Full list of enriched GO terms is provided in Table S4. (H) Left: Example of gained loop hub. Hub over *SRD5A3* (marked 1) was gained at D90 and maintained at D120 (stably gained); hub over *CLOCK* (marked 2) present only at D120. Right: Example of stable loop hub; hub over *SOX2* (marked 3) present D60–D120. In both panels, the upper track shows the compartment score, middle tracks show chromatin loops, and the lower track shows select genes. Orange-highlighted regions indicate hub locations. See also Figure S5, Table S2, and Table S4.

Distal CREs modulate gene expression via chromatin looping,^10,31^ which creates physical interactions at or near the TSS. Using 5-kb Hi-C interaction maps, we detect nearly 30,000 chromatin loops in developing ROs from D60 to D120. Of these, 61.1% are present at least in three time points and 29.2% in all four time points (Figure 5A). Chromatin loops showing interactions with expressed genes are enriched for contacts in promoter regions and depleted in intergenic regions (Figure 5B). Moreover, interaction of expressed genes to other gene promoters tends to increase during development, probably due to the aggregation of promoter regions to form transcription-permissive sub-compartments. In addition, expressed genes with chromatin loops are enriched for protein-coding sequences (Figure 5B).

Chromatin looping is usually distributed in the enhancer and promoter regions, yet many loops may indirectly interact with one another by contact to shared loci and form wider loop networks,^6,72^ including small-scale, isolated cliques or spatially segregated hubs that can control gene expression.^73–75^ To assess gene aggregation, we computed communities defined by genes present in the same 3D space, i.e., genes overlapping or interacting together. The total number of gene communities decreases during RO differentiation, whereas the mean number of expressed genes per community gradually increases (Figure S5A). Gene communities increase in mean size over development (Figure 5C). Notably, communities containing retinal-enriched genes and markers associated with amacrine cells, horizontal cells, and photoreceptors increase in size at D70 and maintain larger sizes throughout differentiation (Figure S5B). These growing communities demonstrate that RO differentiation is accompanied by an increase in genomic *cis* interactions, which may be associated with specific gene expression patterns at the terminal stage. For example, *CNGB1*, a marker gene for differentiated rods, joins a growing community at D90 and is maintained in that community at D120 (Figure 5D). We suggest it is possible that the formation of progressively larger communities via looping form co-regulation networks that reinforce gene identity and stabilize gene expression for differentiated cells in the face of a complex external environment.

We previously demonstrated an enrichment of super enhancers (SEs) for loop contacts in adult retina.^33^ In the developing ROs, we identify ~1,500 chromatin hubs that are enriched for dense loop anchor contacts (see STAR Methods) (Figure S5C). Over 15% of hubs are stably gained and maintained throughout development, whereas relatively few (less than 5%) are stably lost (Figure 5E). We further note that almost no hubs generated from random sets of loops are found at two consecutive time points (Figure 5E). Interestingly, gained and stable loop hubs are more likely to reside in regions that shift toward active A compartments. In contrast, lost loop hubs are more likely to reside in regions consistently shifting toward repressive B compartments (Figure 5F). Genes overlapping with gained loop hubs are mainly enriched for GO terms associated with nervous system development, whereas terms related to eye development tend to appear in the enrichment analysis with genes that overlap with stable hubs, and no term enrichment evident for genes overlapping lost hubs (Figure 5G; Table S4). Thus, the gained hubs are likely involved in fine-tuning neural-related gene expression during RO differentiation, and the hubs involved in retinal cell fate determination are established and could persist in a poised state for activation by other competence factors. As representative examples, a key retina-expressed gene associated with circadian rhythms, *CLOCK*, resides in gained loop hubs on Chr4 with the compartment shift from B to A, whereas *SOX2*, which is progressively silenced during retinal development, is located in a stable loop hub with compartment shift from A to B (Figure 5H). These results suggest that regions with dense looping are particularly critical for lineage specification.

### Genome topology of developing human ROs increasingly resembles that of adult retina

**Figure 6.**
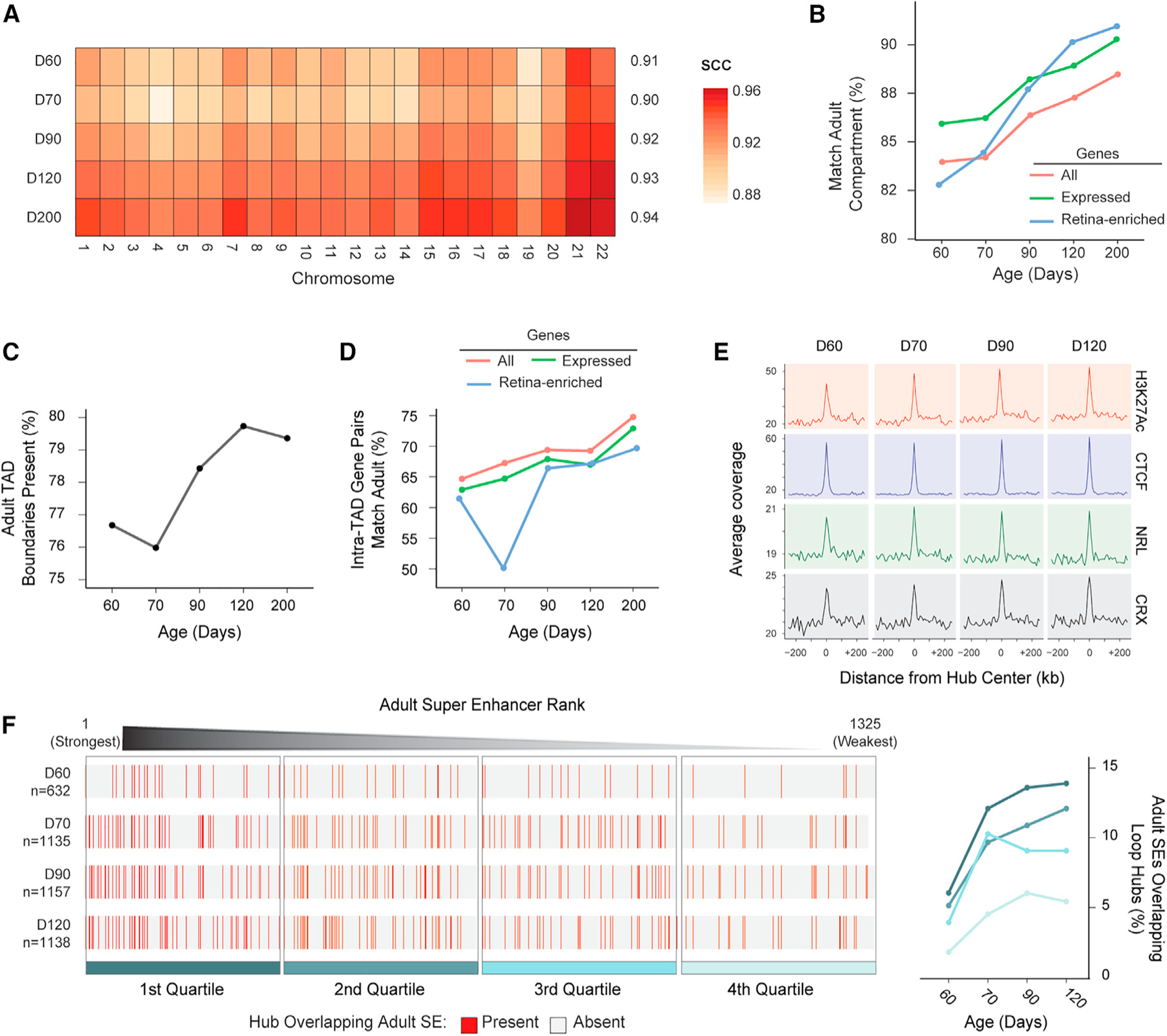
Chromatin structure of developing retinal organoids increasingly resembles that of the adult retina (A) Heatmap of chromatin contact map similarity (SCC value) across chromosomes for each RO differentiation time point compared with adult retina. (B) Percentage of genes in the same compartment type as observed in adult retina by differentiation time points of ROs. (C) Percent of adult TAD boundaries observed in retinal organoids (within ±20 kb). (D) Percent of intra-TAD gene pairs of the adult retina observed in the developing ROs. (E) Enrichment of H3K27Ac, CTCF, NRL, and CRX marks in adult retina around loop hubs called from retinal organoids. (F) Retinal organoid loop hubs overlapping super enhancers (SEs) from adult retina. Left: Adult SEs divided into quartiles by strength. Each row represents a retinal organoid time point. Red lines indicate a hub overlaps a given SE at the specified time point. The total number of hubs called per timepoint is indicated on the far left. Right: The percent of adult SEs with a hub overlapping split by time point and quartile. Color coding matches the quartiles on the left. See also Table S2.

To gain insight into the extent to which differentiating organoids recapitulate *in vivo* retinal development, we compared RO topology with recently reported chromatin architecture in the adult human retina.^33^ Contact maps of developing ROs increasingly resemble those observed in the adult retina as measured by SCC (Figure 6A). Moreover, the percentage of genes located in compartments matching those in adult retina enhances throughout development (Figure 6B). The compartment type of retina-enriched genes appears to dramatically increase between D70 and D90, consistent with the SCC pattern. Similarly, the proportion of adult TAD boundaries present in ROs increases throughout differentiation (Figure 6C), resulting in a progressive increase in number of gene pairs recapitulating the intra-TAD gene pairs observed in adult human retina (Figure 6D). Interestingly, the pattern for gene pairs containing retina-enriched genes diminished sharply at D70 and then restored at D90 with a continuous but slow increase until D200. In the ROs, we also detected an enrichment on loop hubs for all four adult CRE marks (H3K27Ac, CTCF, NRL, and CRX) that are augmented near SE regions in the adult retina, and this enrichment tended to increase gradually during the differentiation process (Figure 6E). The overall proportion of RO loop hubs overlapping with adult retina SEs increases during differentiation, and the strongest SEs are more likely to overlap a hub at all time points (Figure 6F). Thus, loop hubs in ROs and adult retina SEs appear to have similar properties and may perform similar functions during differentiation. Taken together, our results suggest that the chromatin structure is gradually approaching that of the adult retina during RO differentiation. This process is accompanied by changes in the expression of genes localized therein, especially retina-enriched genes, which may be due to chromatin remodeling to form loop hubs and act as SEs to affect gene expression.

### Genome reorganization influences TFs associated with lineage specification

The chronological pattern of developing retina is intrinsically controlled by the dynamic regulation of TF expression, which in turn guides the ability of RPCs to proliferate and produce specific cell types.^20,22,76^ To decipher how 3D structural changes influence development, we focused on a set of TFs known to be involved in retinal cell fate determination to explore the crosstalk between the dynamics of chromatin structure at these TF loci and their expression pattern.

**Figure 7.**
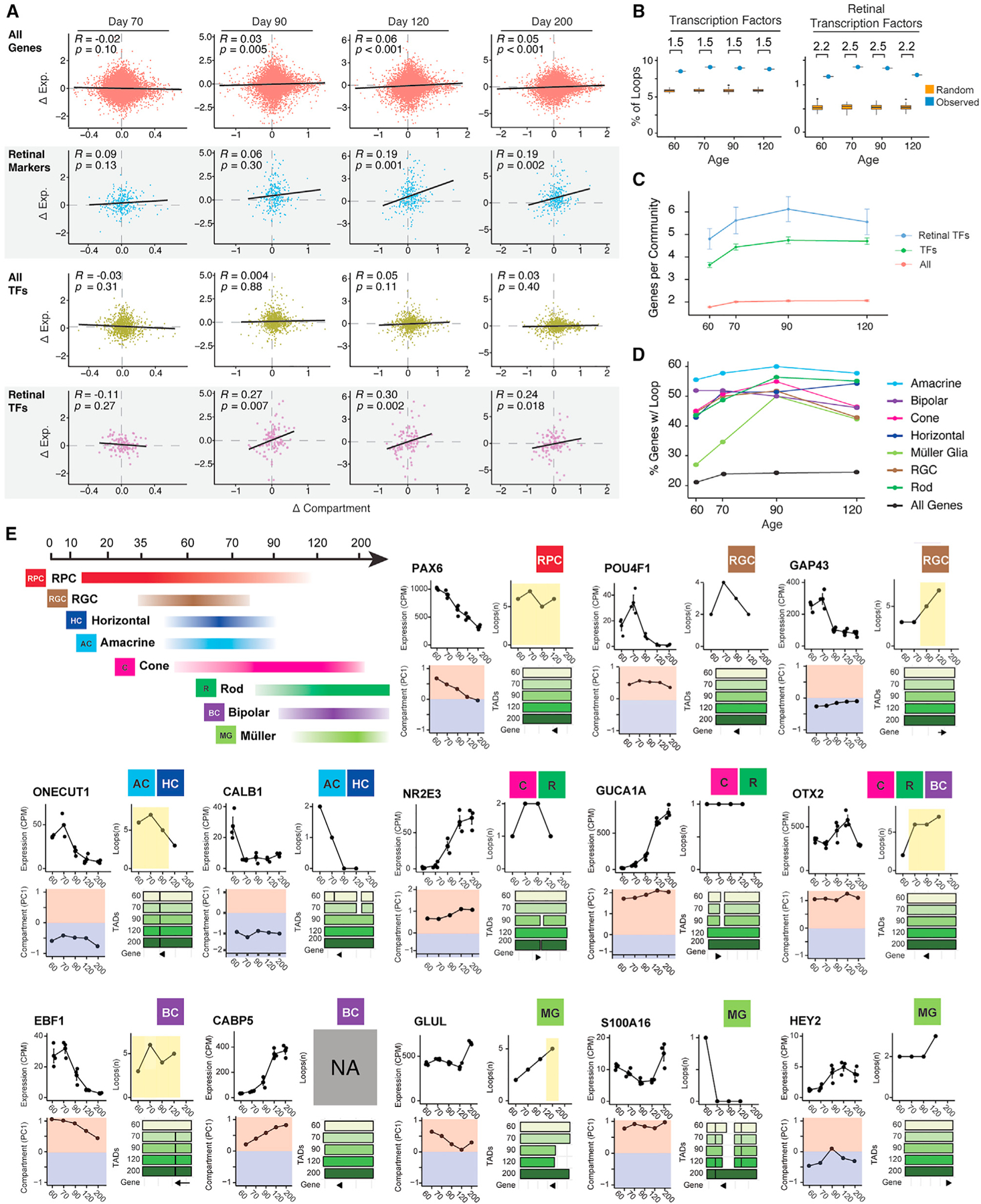
Chromatin structure guides lineage commitment by influencing transcription factor expression in developing ROs (A) Correlation between expression and compartment changes relative to D60 for all genes (top row), retina-enriched genes (second row), all transcription factor (TF) coding genes (third row), and for retina-associated TF coding genes (bottom row). Spearman’s *R* value and p value are shown. (B) Proportion of loops in contact with at least one transcription factor coding gene (left) or retinal transcription factor coding gene (right). The values listed above each pair of points indicate the enrichment of the real value compared to the mean value of the 100 shuffled loop sets. Boxplots represent the distribution of shuffled loop sets showing the median and interquartile range (IQR); whiskers mark 1.5x the IQR; data beyond 1.5x the IQR are plotted as individual points. (C) The number of all genes, TF coding genes, and retinal-associated TF coding genes found per gene community. Bars represent the standard error. (D) Percentage of cell-type marker genes overlapping with loop anchors across differentiation time points of developing ROs. (E) Overview of cell-type development in retinal organoids (top left). The remaining subpanels show changes in genome topology and expression for select retinal marker and TF coding genes. In each gene subpanel, top left shows expression in CPM. Top right shows the number of loops overlapping the gene with yellow highlights when a hub overlaps the gene. Bottom left shows the mean compartment score across the gene. Bottom right shows TADs overlapping the gene and gene direction (arrow). Cell types associated with each gene are shown in the top right of each subpanel. Topology and expression data are available in tabular form for all genes in Table S5. See also Table S2 and Table S5.

To examine whether changes in 3D gene structure affect changes in gene expression during retinal organoid differentiation, we calculated the correlation between changes in compartment score and changes in gene expression relative to D60 (Figure 7A). We find an extremely weak (*R* range: 0.03–0.05) correlation between these compartment and expression changes. Similarly, we observed no significant correlation between compartment and expression changes among all TF coding genes. In contrast, we observe a moderate positive correlation in retinal TF coding genes (*R* range: 0.24–0.30) and retinal marker genes (*R* = 0.19). This correlation appears first in retinal TFs (D90) and then later in retinal cell-type marker genes (D90). This pattern is consistent with a model where changes in expression and genome topology for retinal TFs influence the subsequent opening of chromatin structure around marker genes associated with retinal cell types.

Genomic looping also appears to contribute to cell-type development. Retinal TFs exhibit a >2 enrichment in loop contacts compared with null expectation and non-retinal TFs (Figure 7B). These loops form gene communities and communities containing retinal TFs are larger on average at all time points than other communities (Figure 7C). Gene communities are largest at D90, corresponding to the time point where the greatest proportion of retinal cell-type marker genes are interacting with loops (Figure 7D).

While we observe coordinated changes in compartment score, loop contacts, and expression at the macro scale, the correlations are highly heterogeneous at the level of individual genes encoding retinal TFs and cell-type markers (Figure 7E; Table S5). For example, *PAX6, NR2E3, EBF1, CABP5, GLUL*, and *HEY2* all show changes in gene expression corresponding to similar changes in compartment score but *POU4F1* and *CALB1* change expression without compartment score changes. We observe that *GAP43* decreases expression while gaining a loop hub at D90 but *ONECUT1* decreases expression before losing a loop hub at D120. Similarly, *CALB1* decreases expression alongside the formation of a new TAD boundary at D70 and *GUCA1A* increases expression as it loses a nearby TAD boundary but *S100A16* increases expression after gaining a TAD boundary. Thus, taken together, our results suggest a complex relationship between 3D genome topology and regulation of gene expression throughout retinal development. Our results serve as a broad atlas cataloging these changes to serve as a resource while dissecting the relative contributions of multi-level changes in genomic structure on gene expression throughout retinal development.

## DISCUSSION

Despite broad anatomical and physiological similarities, animal models do not faithfully reflect temporal underpinnings of developmental states and complexities of the human retina.^28,42,43,77^ Here, we took advantage of organoid cultures that recapitulate human retinal development and disease to dissect the dynamics of genome topology at specific stages of cellular specification. We constructed high-resolution contact maps and reveal characteristics of chromatin landscape at distinct genomic scales and its association with altered gene expression. Our results also imply that hierarchical levels of 3D genomic structures are involved in establishing and maintaining the spatial landscape required for retinal lineage commitment, thereby participating in retinal differentiation in a well-orchestrated temporal manner. By integrating transcriptomic data, we highlight the role of chromatin dynamics in retinal cell fate commitment at key TF coding genes. Notably, genome topology of developing organoids increasingly resembles that of adult retina over time, further validating the use of organoids for investigating dynamic changes in genomic regulation during retinal development.

Since their initial identification, euchromatin and heterochromatin have been broadly characterized as active and inactive regions of the genome, respectively.^78^ In accordance, Hi-C studies have consistently observed enrichment of expressed genes and active epigenetic marks in A compartment (i.e., euchromatin) relative to B compartment (i.e., heterochromatin).^11,34,51^ Genome compartmentalization is dynamic throughout lineage specification and regions undergoing compartment changes are often enriched for DE genes^11,79,80^; however, compartment changes in this study do not parallel expression changes for most genes, consistent with previous reports showing no change in expression of a majority of genes that switch compartments during pig spermatogonia or mouse midbrain differentiation.^81,82^ Furthermore, we observed that most changes in gene expression occur without compartment changes. Similarly, most changes in gene expression occur in stable compartments during differentiation of mouse dendritic cells^83^ and during sex differentiation in the Barbados nut tree.^84^ Thus, although compartments are globally associated with gene expression, compartment switch and gene expression changes are correlated only at a subset of loci. We suggest that higher compartment resolution^85^ and/or additional subdivision of A and B compartments based on epigenetic marks^10^ may be necessary to resolve the relationship between compartment and gene expression.

Early Hi-C studies indicated conservation of TADs across cell types and species^10,11,58,86^; however, a more detailed examination of 21 mature human cell and tissue types identified ~30% of TADs in fewer than half,^87^ implying that TAD borders shift during cell-type specification and differentiation. Indeed, several reports show altered TAD borders during differentiation of multiple cell types.^80,82,88^ Our results also demonstrate a change in nearly 40% of TAD borders in at least one differentiation state, likely reflecting maturation of cell types as well as changes in cell population in developing organoids. In concordance, we identify enrichment of stable TAD boundaries for genes involved in housekeeping functions and association of dynamic borders with cell-type and development stage-specific functions. We therefore propose that lineage-specific TAD boundaries play a key role in establishing cell identity.

We noted that TADs in A compartment are smaller than those in B compartment, likely due to the presence of more CREs and coding genes in active compartment A regions,^33,58^ leading to more frequent intra-TAD interactions compared with the inter-TAD interactions and making them easily separated from each other in proximity region. In contrast, in the repressive B compartment regions, larger regional intra-TAD interactions may be required to separate with more distant and weaker inter-TAD interactions because of the lack of sufficient interaction frequency differences between proximal regions.

The majority of TADs participating in higher-order interactions like TAD cliques are reportedly present in B compartment, and the proportion increases as differentiation proceeds.^12,81,89,90^ Predictably, genes residing in these primarily B compartment TAD cliques exhibit lower expression relative to genes in non-clique TADs.^12,81,90^ Using the clique identification method described in these prior studies, we observe that ~75% of TAD cliques in our study are present in A compartment and this proportion remains stable throughout development. Our results are consistent with another study identifying ~30% of TADs in B compartment across four human cell lines^68^; yet, unlike in our study, TAD cliques across the human cell lines still contained genes with lower expression than in non-TAD cliques.^68^ We also noted an enrichment of SINEs and a deficit of LINEs in TAD cliques of our ROs in contrast to enhanced LINEs in TAD cliques from all four human cell lines. We conclude that properties of TAD cliques are cell-type- and lineage-specific and that additional data would be necessary before one can confidently ascertain general principles of TAD clique properties.

During retinogenesis, sequential and spatiotemporally restricted activation of TFs contributes to generation of diverse cell types from RPCs.^91^ Furthermore, the combined TFs form a complex regulation network maintaining different cell types of the retina.^92^ Typically, genome topology and transcription are dynamically linked. Gene expression can take precedence or lag changes in chromatin structure. During cell reprogramming, it is more often the case that compartment switch precedes changes in gene expression, including around pluripotency TFs.^66^ In our study, temporal expression pattern of retinal TFs is closely associated with changes in the chromatin structure. For example, *PAX6*, an essential paired- and homeobox-containing TF expressed in RPCs,^93–95^ shows concurrent decrease in gene expression and compartment score during differentiation. *PTF1A*, a bHLH TF required for horizontal and amacrine cell fate specification in retina,^96^ exhibits a relatively high expression in early stage of differentiation when in the active A compartment, but its expression levels are gradually downregulated at all subsequent time points by switching to the repressive B compartment. Notably, we only observe a significant correlation between TF expression and compartmental changes when focusing on retinal TFs and not when all expressed TFs are considered. Given the diversity of cell types and increased complexity of tissues throughout development, the relationship between gene expression and 3D genome may not be evident due to excessive noise caused by mixed signals from bulk pools containing cell-type-specific topologies near TFs, especially at later time points.

We realize that combined action of a select group of TFs, and not the expression of a single factor, is crucial for determining a specific cell fate during mammalian retina development. Combinatorial function of TFs creates a quantitatively precise, controlled, and complex pattern of gene regulatory relationships across different retinal cell types, ensuring consistency and robustness in the differentiation process.^22^ In our study, we uncover an interacting regulatory network of the retinal TF coding loci in close spatial proximity with more loop formation. Moreover, communities containing retinal TFs are larger than those without TFs. Additionally, TF-containing TADs form more cliques. The enrichment of TFs within these higher-order genomic structures suggests that establishment of multi-loci regulatory networks contribute to precise regulation of retina TFs, consistent with the hypothesis that TFs require a topologically permissive environment to drive lineage-specific expression.

Most of the susceptibility variants identified in GWASs are present in non-coding regions and predicted to influence gene expression.^97,98^ Moreover, these variant regions tend to harbor CREs with *a priori* unanticipated, promiscuous, and context-dependent behavior, which makes it difficult to link to their target genes.^67^ Using a high-resolution Hi-C map of the adult human retina, we have previously been able to link several associated loci to retinal diseases.^33^ However, many non-coding variants may impact gene regulation during development. Using iPSC-derived human ROs, we have been able to detect not only dynamic changes in chromatin architecture at different stages of development but also can predict the link of disease-associated variants to target genes without requiring patient samples. We note the two excellent examples of such studies that demonstrate altered genome structure by retinal disease-associated variants.^32,99^ In addition to providing unique insights into genomic regulation especially during human retinal development, our Hi-C studies should advance understanding of the non-coding genome relevant to both Mendelian as well as complex retinal traits.

### Limitations of the study

Although ROs can serve as a useful model for human retinal development and recapitulate primary developmental states, these cultures do not fully reflect the cell-type proportions or perfect stratification and synaptic organization within the human retina. Additionally, the organoids do not develop fovea/macula and therefore do not capture relevant developmental events precisely. However, given the limitations on fetal tissue, ROs represent the best available model system for investigating early retinal development.

We note that retinal development, *in vivo* and in organoids, involves substantial changes in cellular composition during differentiation. Our bulk Hi-C data capture the average genome topology across cell types. Therefore, these data are most informative for understanding topological changes near marker genes for high-abundance cell types (such as photoreceptors, ON-bipolar cells, Müller glia) and within genomic regions that are not cell type specific. We eagerly anticipate future optimization of organoid culture and single-cell Hi-C methods to fully disentangle the effects of changes in cell composition from changes in genomic architecture within cell types.

Despite these limitations, our dataset and analyses represent the most complete characterization to date of the dynamic 3D genome during RO differentiation and serve as a roadmap for future studies. These results provide critical insights into the epigenetic landscape of the developing human retina, especially for the most abundant cell types that are commonly associated with vision impairment in retinal degeneration.

## STAR★METHODS

### RESOURCE AVAILABILITY

#### Lead contact

Further information and requests for resources and reagents should be directed to and will be fulfilled by the lead contact, Anand Swaroop (swaroopa@nei.nih.gov).

#### Materials availability

All unique/stable reagents generated in this study are available with a completed Materials Transfer Agreement per NIH policy. Further information and requests for resources and materials should be directed to and will be fulfilled by the lead contact, Anand Swaroop (swaroopa@nei.nih.gov).

#### Data and code availability

- All datasets used are summarized in the key resources table. The next generation sequencing data generated in this study are available at the Gene Expression Omnibus (GEO, accession number: GSE229683).
- This paper does not report original code.
- Any additional information required to reanalyze the data reported in this work paper is available from the Lead Contact upon request.

### EXPERIMENTAL MODEL AND STUDY PARTICIPANT DETAILS

#### Cell lines

The human embryonic stem cell (ESC) line H9 (female) was maintained under a feeder-independent condition according to the WiCell protocol (https://www.wicell.org/product-files/cultureProtocols/SH-2.pdf). Cells were cultured in mTeSR1 medium (Stem Cells Tech) on hESC-qualified matrix (Matrigel, Corning)-coated plates with daily medium change. The cells were routinely passaged every 3 to 5 days by dissociation with EDTA. Rock inhibitor was added to the new passaged cell medium to prevent dissociation-induced apoptosis.

#### Differentiation of retinal organoids

Retinal organoids were differentiated following a protocol as previously described^37,49^ with minor modifications. Briefly, on day 0 (termed as D0) of differentiation, 90% confluent human ESCs in one well of 6-well plate were detached by 5 mM EDTA. Cells were dissociated into small clumps by pipetting gently and were then resuspended in 9 mL mTeSR1 medium containing 10 mM ROCK inhibitor (Y027632, TOCRIS) in a 90-mm ultra-low-attachment dish (S-bio, PrimeSurface Dish90, Cat#MS-90900Z). From D1 to D3, culture medium was gradually transitioned to neural induction medium (NIM) containing DMEM/F12 (1:1) and supplemented with 1% N2 supplement (ThermoFisher Scientific), 0.1 mM nonessential amino acids (NEAA, ThermoFisher Scientific) and 2 μg/mL heparin. On D7, Cell aggregates were then seeded onto three wells of growth-factor-reduced (GFR) Matrigel-coated 6-well plate for adherent culture. On D16, the culture medium was switched to DMEM/F12 (3:1 NIM) and supplemented with 2% B27 (without vitamin A, ThermoFisher Scientific), 0.1 mM NEAA and 1% antibiotic–antimycotic (ThermoFisher Scientific) and changed daily.

From D28 to D42, horseshoe-shaped neural retinal domains were manually detached with glass needle with curved tip under inverted microscope, transferred to each well of ultra-low attachment round bottom 96-well microplate (S-bio, PrimeSurface Dish90, Cat#MS-9096UZ) and cultured in suspension with 3:1 NIM plus 20 ng/mL IGF-1 (Invitrogen). At D42, 10% (v/v concentration) fetal bovine serum (FBS, R&D Systems) and 100 μM Taurine (Sigma) were added to the culture medium. 9-cis Retinal were added to the differentiation medium from D63 with a final concentration of 1 μM and was subsequently reduced to 0.5 μM from D92. Medium were changed every 2–3 days.

### METHOD DETAILS

#### Hi-C experiment and library preparation

Hi-C was performed on 8–10 organoids from each time point according to the manufacturer’s instruction of Arima Genomics. In brief, organoids were dissociated to single cell following an optimized protocol of papain system^130^ and then crosslinked with 2% para-formaldehyde (PFA, FDNeuroTechnologies) for 10 min. Subsequently, the crosslinked samples were quenched by 125 mM glycine for 5 min and frozen until use at −80° C until use for Hi-C experiments. The detailed experimental procedures of Hi-C were performed as described.^33^

#### RNA extraction and library preparation

Total RNA was extract from 3 to 4 pooled frozen organoids per sample using the RNeasy Mini kit (Qiagen) according to manufacturer’s protocol. Concentration and quality were evaluated with the Agilent 2100 Bioanalyzer system (Agilent Tech). RNA samples with RIN more than 7 were utilized for library generation using TruSeq Library Preparation Kit (Illumina Inc.).

#### Immunofluorescence staining

Retinal organoids were collected at appropriate time points throughout the differentiation procedure. Immunofluorescence staining was performed as described previously^49^ with slight modifications. In brief, 3–4 organoids per sample were fixed with 2% PFA in PBS for at least 30 min at room temperature. Fixed organoids were dehydrated in a sucrose gradient from 10% to 30%, then embedded in optimal cutting temperature compound (VMR) and frozen at −80° C. Cryosections were generated using a cryostat (Leica Biosystems) at 10 μm thickness. Sections were dried on Superfrost Plus glass slides (Fisherbrand) and stored at −20° C until use.

For immunostaining, slides were removed from freezer and warmed at room temperature for 10–15 min. Samples were hydrated with PBS for 10 min and then permeated with 0.2% Triton X-100 (Sigma-Aldrich) for 15 min. After permeation, sections were washed with PBS for 5 min and followed by blocking with 10% donkey serum (Gibco) in PBS (termed as blocking buffer) for minimum of 1 h at room temperature. Then, sections were immediately incubated with primary antibodies at 4° C overnight. Both primary and secondary antibodies are diluted to the appropriate concentration (listed in the key resources table) in blocking buffer according to manufacturer’s specification sheet. After three 5-min washes in PBS, secondary antibodies were added to samples for 2 h. Sections were washed with PBS for 5 min, incubated with DAPI for 10 min and washed with PBS again for 5 min. Subsequently, samples were mounted with Fluormount-G mounting medium and covered with coverslips. Fluorescence images were acquired on a Zeiss LSM700 confocal microscope system and exported with ZEN software. Images are processed and quantification with Adobe Photoshop 2022, Adobe Illustrator 2022, and ImageJ v2.3.0. At least three independent organoids were included for quantification.

### QUANTIFICATION AND STATISTICAL ANALYSIS

#### RNA-seq analysis

RNA-seq data analysis was performed as previously described.^49^ In brief, paired-end sequencing was performed to 125 bases on Illumina HiSeq 2500. The alignment and quantification pipeline used human reference genome GRCh38.p7 and Ensembl v102 for annotation. Transcript-level counts were performed by kallisto^118^ v0.45.0 package and summarized to gene level. Gene level counts were converted to count per million (CPM) and then TMM normalized using the edgeR^131^ v.3.32.1 package in R (v.4.0.3)/Bioconductor environment. Gene enrichment analyses were performed using gProfiler2 v0.2.1,^132^ ShinyGO^133^ v0.76, and clusterProfiler.^121^ PCA plots were generated using PCAtools v2.10.0. Heatmaps were generated using ComplexHeatmap v2.14.0.^122^ The proportion of cell types throughout development was roughly estimated by calculating the change in expression of retinal cell type marker genes across differentiation.

#### Hi-C data processing

Hi-C data pre-processsing and analysis was carried out as described previously^33^ with minor modifications. Briefly, raw sequencing data of *in situ* Hi-C were processed using HiCUP^102^ to truncate, map and filter the reads and to generate a quality assessment using an Arima-specific *in silico* digested hg38 genome for the retinal organoid samples. Subsequently, filtered bam files generated from HiCUP were then converted to.hic files^115^ and to Homer^106,134^ tag directories. Resolutions of Hi-C were estimated using HiCRes.^101^ Reproducibility between samples has been assessed using SCC calculated by HiCRep^50^; for each pair of datasets, the SCC has been calculated by chromosome then averaged value across all autosomes. For comparison with human retina, the SCC has been calculated between the human retina and each sample for each chromosome using HiCRep.^50^ Contact maps were plotted using Juicebox^115,116^ (Figure 1F), CoolBox^108^ (Figure 1G), matplotlib^53,128^ (Figure S1C), and the WashU Epigenome Browser^112^ (Figure 4D).

#### Gene lists and genomic regions of interest

Several gene lists are used throughout analyses in this manuscript. All genes indicate every gene in Ensembl 102 while expressed genes represents just the subset with at least 1 CPM expression in all samples in at least one time point. Retina-enriched genes were downloaded from the Human Protein Atlas (proteinatlas.org). Transcription factor coding genes were extracted from Lambert et al.^100^ and the subset of retinal TFs were selected based upon manual curation of this list. Retinal cell type marker gene lists were generated by combining manually curated genes with markers derived from previously published scRNA-seq data.^46^ Briefly, we imported the data into Seurat v4.3.0.1^129^ and executed the FindMarkers command for each cell type. Markers detected in at least 75% of the target cell type and in <25% of all other cell types were retained (up to n = 50). The location of protein coding and ncRNA was extracted from Ensembl v102; ncRNA includes all genes labeled as miRNA, miscRNA, rRNA, snRNA, snoRNA, vault RNA, lncRNA, scRNA, sRNA, or scaRNA. Genomic repeats were extracted from the hg38 genome assembly repeat mask file from UCSC genome browser.

#### Contacts enrichment at gene TSSs

Contact enrichment at the MANE TSS site for each gene was calculated genome-wide using HiCmapTools.^114^ Enrichment values at TSS of non-expressed (<1 CPM), expressed (≥1 CPM), or at retina-enriched genes identified by the Human Protein Atlas (Table S2) were computed for each sample. For each gene, permutation testing was performed by HiCmapTools by comparing the observed contact counts to a distribution of 1000 contact counts randomly extracted from the same chromosome; genes with a normalized rank <0.05 by this method were identified as significantly enriched. The percent of genes with significant contact enrichment at each time point was calculated by dividing this value by the total number of genes per category.

#### Compartments

Compartments have been called with HOMER^106^ using 100 kb sliding windows with a step of 50 kb. Correlation between compartments in different samples has been computed using R. Principal component analysis of compartment score between samples was performed using PCAtools v2.10.1. Stably switching compartments have been identified as 50 kb genomic bins that switch PC1 signs just once throughout development (e.g., PC1 < 0 at D60 and D70, PC1 > 0 D90, D120, and D200). The percentage of expressed and retina-enriched genes in region with stable or switching compartments has been computed using R. For the saddle plots (Figure 2B), each chromosome arm was sorted into 40 equal bins based on the compartment score and their interactions were quantified then distance normalized with GENOVA.^103^ In each panel of saddle plots, interactions in the bottom left corner represent interactions between B compartments and interactions in the top right corner represent A–A interactions. Compartmentalization strength is calculated as the mean of the 5 by 5 blocks at each corner plot representing enrichment of either homotypic (A–A and B–B) or heterotypic (A–B) interactions. Significant changes in compartment strength were identified using dcHiC run with the default settings^56^ then visualized as circos plots using the circlize package.^123^ GREAT^57^ analysis was performed on genes present in regions with a Mahalanobis distance >1 using the default settings. Sankey plots of compartments were generated using ggalluvial.^127^ Specific compartment change examples were plotted using IGV.

#### Comparison of compartment changes to gene expression

We identified genes which steadily increased or decreased expression across all time points, then ranked genes by the ratio of expression at D200 over D60. We retained the highest and lowest 10 percent of genes and checked what percent overlapped compartments that shifted toward A or toward B across differentiation. For Figure 3D, all genomic bins which stably switched compartments were clustered and plotted using ComplexHeatmap v2.14.0. We counted the number of differentially expressed genes between D60 and D200 (p < 0.05; fold change >1.5) that overlap stably switched compartments and identified significant GO terms among these genes using gprofiler2. Example plots showing changes in compartment score and expression were plotted using IGV. In Figure S3A, expressed genes and retina-enriched genes were divided into groups based on the compartment type overlapping their MANE select TSS. Observed results were compared to 100 sets of random compartment scores generated by shuffling the observed compartment scores across the genome. For all genes, retinal marker genes, TFs, and retinal TFs we calculated the Spearman correlation between compartment score changes (D200-D60) and changes in expression (D200-D60 in log2(CPM) format) using R.

#### TADs

TAD calling has been performed at each time point using DomainCaller^58^ at a 10kb resolution. Stable TAD boundaries were identified as boundary locations that exist across all time points (±20 kb). Stably gained TAD boundaries were defined as boundaries absent at D60 but identified at a later time point and then present at all subsequent timepoints. Conversely, stably lost boundaries were defined as TAD boundaries present at D60, lost at some later time point, then absent in all subsequent time points. Boundary genes were defined as all expressed genes with a TSS located within 10kb of a TAD boundary while all other genes were defined as non-boundary.

Boundary genes near stable, gained, or lost TAD boundaries were used to identify enriched ontological terms with ShinyGO v0.76.3. Insulation scores were calculated with the method of summing up contacts in a sliding diamond-shaped window along the corners on the main diagonal of the Hi-C matrix.^135^ Domain scores were computed as described previously.^58^ Briefly, contact counts were extracted from mcool files using GENOVA^103^ v1.0.1 at 10 kb resolution. Then, the total number of intra-TAD contacts (i.e., those within a single focal TAD) were divided by the number of inter-TAD contacts (i.e., the total number of contacts between the focal TAD and all other intra-chromosomal TADs). Changes in domain score were clustered using clusterProfiler^121^ and enriched GO terms for genes within these clusters were identified using the enrichGO function in this package. TAD cliques were defined as described previously.^68^ Briefly, significant TAD-TAD interactions were found using the NCHG^109^ tool with the default settings then TAD cliques were identified via the igraph^110^ R package. 100 sets of random TAD cliques were generated by shuffling the size of cliques among the TADs.

#### Chromatin loops

Loops have been called at each time point using Mustache^136^ at a 5 kb resolution with an FDR threshold of 0.05. 100 sets of random loops were generated by shuffling the bedpe file using bedtools^105^ shuffle.

#### Gene-interacting regions

We computed the gene-interacting regions by considering expressed-gene overlapping organoid loops (or 100 random loop sets) at each time point. From these loops sets, the foot not overlapping in the gene was annotated using HOMER. The enrichment was defined as the log2 ratio of the number of unique expressed-gene/chromatin annotation pairs over the total number of annotated regions. Further, we found the percent of loops which overlapped a TF coding gene or retinal TF coding gene and compared these results to 100 randomly generated loop sets.

#### Gene networks

We computed the organoid gene networks by integrating 5 kb resolution loops and genes, associated to their expression level, at each time point. We identified gene communities as assemblages of genes which either overlapped and/or interacted through chromatin loops. Communities with more than one locus (gene or loop foot) were considered for further analysis.

#### Loop hubs

We computed the total number of loop feet coverage using 30 kb genomic windows with a step of 3kb using bedtools.^105^ For each sample, we selected the windows with a minimum coverage of 5 loop feet and stitched them together allowing a gap of up to 30 kb between two windows using bedtools. We then merged these regions across samples and, for each sample, computed the total number of loop feet covering each region. For each sample, we selected the regions overlapping with at least 5 loop feet as a hub for further analysis. To assess how hubs compare to adult retina features, they have been overlapped with adult retinal SEs and their overlap has been computed depending on the adult retina SE ranks.^33^ The average coverage in adult retina for selected chromatin marks and factors^33^ have been assessed at hubs using HOMER.^106^ Dynamic hubs have been called by categorizing hubs as stable (present at each time point), gained (present at D120 and stably gained from any given time point after D60), lost (present at D60 and stably lost from any given time point before D120) and others (other possible configurations). Hub-overlapping genes have been defined as any organoid expressed gene (gene expressed at any time point), for which at least a part of the gene body overlap with a hub. The genes overlapping gained and lost hubs have been processed using Panther, to perform statistical overrepresentation test using the category GO biological processes complete, using all human genes as control. To assess the compartment dynamic versus hub dynamic, the average PC1 values at gained, lost and stable hubs have been computed. Hubs have been categorised as “stable increase of PC1” (PC1 value is higher at D200 compared to D060 and for each other pair of samples, the PC1 value of the latest time point is higher or equal to the PC1 value of the other time point) and “stable decrease of PC1” (PC1 value is lower at D200 compared to D060 and for each other pair of samples, the PC1 value of the latest time point is lower or equal to the PC1 value of the other time point).

#### Comparison of RO Hi-C data with that of the adult retina

Adult retinal Hi-C and epigenetic data was taken from our previously published work.^33^ Comparisons with adult compartments were completed by performing SCC as described above and quantifying the number of 50kb windows with A/B compartment assignments matching adult by time point. For each time point, the percentage of genes present in the same compartment as in the adult retina were computed for all genes, expressed genes, and retina-enriched genes lists created as described above. The percentage of adult TADs boundary present at each time point was assessed by identifying all overlapping boundaries (±20 kb) between the adult retina and each time point using GenomicRanges. The percentage of adult gene pairs also present in the same TADs at a given time point was assessed for all genes, expressed genes, and retina-enriched genes lists created as described above. Hubs were checked for overlap with adult super enhancer locations from^33^ using GenomicRanges.

#### Statistics

Quantifications were performed on data obtained from at least 3 individually cultured organoids in separate wells of 96-well plate from 3 independent differentiation batches unless otherwise indicated. Statistical comparisons were analyzed by Student’s *t* test (2 sided) or one-way ANOVA with Dunnett’s post hoc test on R (v4.1). *p* value less than 0.05 was considered statistically significant differences; ns: not significant, *p < 0.05, **p < 0.01, ***p < 0.001, and ****p < 0.0001. Data visualizations were performed using ComplexHeatmap,^122^ ggplot2,^124^ and the ggpubr^137^ R packages. Hi-C data was visualized using the UCSC Genome Browser,^111^ Juicebox,^116^ and IGV.^117^

## Supplementary Material

1

2

3

4

5

6

## Figures and Tables

**Table:** KEY RESOURCES TABLE

REAGENT or RESOURCE	SOURCE	IDENTIFIER
Antibodies		
Mouse monoclonal anti-PAX6 (1:100)	DSHB	Cat#pax6; RRID: AB_528427
Sheep polyclonal anti-CHX10 (VSX2, 1:200)	Abcam	Cat#ab16142; RRID: AB_302279
Mouse monoclonal anti-RHO (1:400)	Millipore	Cat#MAB5356; RRID: AB_2178961
Rebbit polyclonal anti-OPN1LW (1:400)	Millipore	Cat#AB5405; RRID: AB_177456
Mouse monoclonal anti-BRN3A (1:200)	Millipore	Cat#MAB1585; RRID: AB_94166
Mouse monoclonal anti-PCNA (1:400)	Abcam	Cat#ab29; RRID: AB_303394
Rabbit polyclonal anti-Lamin A/C (1:200)	Invitrogen	Cat#PA1–41104; RRID: AB_2265742
Rabbit polyclonal anti-PROX1 (1:200)	Abcam	Cat#ab11941; RRID: AB_298722
Goat polyclonal anti-NRL (1:200)	R&D Systems	Cat#AF2945; RRID: AB_2155098
Donkey anti-sheep IgG conjugated Alexa Fluro 568 (1:500)	Thermo Fisher	Cat#A-21099; RRID: AB_2535753
Donkey anti-mouse IgG conjugated Alexa Fluro 568 (1:500)	Thermo Fisher	Cat#A-10037; RRID: AB_2534013
Donkey anti-mouse IgG conjugated Alexa Fluro 488 (1:500)	Thermo Fisher	Cat#A-21202; RRID: AB_141607
Donkey anti-rabbit IgG conjugated Alexa Fluro 568 (1:500)	Thermo Fisher	Cat#A-10042; RRID: AB_2534017
Donkey anti-rabbit IgG conjugated Alexa Fluro 488 (1:500)	Thermo Fisher	Cat#A-21206; RRID: AB_2535792
Donkey anti-goat IgG conjugated Alexa Fluro 555 (1:500)	Thermo Fisher	Cat#A-21432; RRID: AB_2535853
Chemicals, peptides, and recombinant proteins		
Dulbecco’s Modification of Eagle’s Medium (DMEM)	ThermoFisher	Cat#11995065
Ham’s F12 Nutrient Mix	ThermoFisher	Cat#11765054
DMEM/F12 + GlutaMAX	ThermoFisher	Cat#10565018
B-27^™^ Supplement (50X), minus vitamin A	ThermoFisher	Cat#12587010
N-2 Supplement (100X)	ThermoFisher	Cat#17502048
Human IGF-I Recombinant Protein	ThermoFisher	Cat#RP-10931
Rock inhibitor (Y-27632)	TOCRIS	Cat#1254
mTeSR^™^1 medium	STEM CELLTech	Cat#85850
Fetal Bovine Serum - Premium Select	R&D Systems	Cat#S11550
GlutaMAX^™^ Supplement	ThermoFisher	Cat#35050061
MEM Non-essential Amino Acid Solution (100X)	ThermoFisher	Cat#11140050
Antibiotic-Antimycotic (100X)	Gibco	Cat#15240112
Heparin	Sigma-Aldrich	Cat#H3149
EDTA	Quality Biological	Cat#351-027-101
hESC-qualified matrix Matrigel^®^	Corning	Cat#354277
Matrigel^®^ Growth Factor Reduced (GFR)	Corning	Cat#354230
Taurine	Sigma-Aldrich	Cat#T8691
9-cis-Retinal	Sigma-Aldrich	Cat#R5754
DMSO	Sigma-Aldrich	Cat#D4540
Papain	Worthington Biochemical	Cat#LS003119
Critical commercial assays		
Click-iT^™^ EdU Cell Proliferation Kit, Alexa Fluor^™^ 488	ThermoFisher	Cat# C10337
Arima-HiC Kit	Arima Genomics	Cat# A510008
RNeasy Mini Kit	Qiagen	Cat# 74104
TruSeq RNA Library Prep Kit v2	Illumina	Cat# RS-122–2001
Deposited data		
Hi-C DNA sequencing	This paper	GEO:GSE229681
bulk RNA-sequencing	This paper	GEO:GSE229682
Hi-C DNA sequencing (adult human retina)	Marchal et al.^33^	GEO:GSE202471
Human transcription factors	Lambert et al.^100^	http://humantfs.ccbr.utoronto.ca/
H3K27ac (SRR10172858), CTCF (SRR10172909), NRL (SRR10172897) and CRX (SRR10172903) ChIP-seq (human retina)	Aldiri et al.^30^	GEO: GSE87064
Experimental models: Cell lines		
human ES cell line H9	WiCell	Cat#WA09
Software and algorithms		
R version 4.0–4.2	The R project	https://www.r-project.org/
Bioconductor	Bioconductor	https://www.bioconductor.org
GREAT v4.0.4	McLean et al.^57^	http://great.stanford.edu/public/html/
HiCRep	Yang et al.^50^	https://github.com/TaoYang-dev/hicrep
HiCRes	Marchal et al.^101^	https://github.com/ClaireMarchal/HiCRes
HiCUP	Wingett et al.^102^	https://www.bioinformatics.babraham.ac.uk/projects/hicup/
GENOVA	van der Weide et al.^103^	https://github.com/robinweide/GENOVA
dcHiC	Chakraborty et al.^56^	https://github.com/ay-lab/dcHiC
DomainCaller	Dixon et al.^58^	https://github.com/XiaoTaoWang/domaincaller
gProfiler	Raudvere et al.^62^	https://biit.cs.ut.ee/gprofiler/page/citing
Chrome3D	Paulsen et al.^104^	https://github.com/Chrom3D/Chrom3D
Bedtools	Quinlan et al.^105^	https://bedtools.readthedocs.io/
HOMER	Heinz et al.^106^	http://homer.ucsd.edu/homer/index.html
cooltools	Abdennur et al.^107^	https://cooltools.readthedocs.io/en/latest/index.html
CoolBox	Xu et al.^108^	https://github.com/GangCaoLab/CoolBox
NCHG	Paulsen et al.^109^	https://github.com/Chrom3D/Chrom3D
igraph	Csárdi et al.^110^	https://igraph.org/r/
UCSC genome browser	Kent et al.^111^	http://www.genome.ucsc.edu/
WashU Epigenome Browser	Li et al.^112^	http://epigenomegateway.wustl.edu/browser/
GenomicRanges	Lawrence et al.^113^	https://bioconductor.org/packages/release/bioc/html/GenomicRanges.html
HiCmapTools	Chang et al.^114^	https://github.com/changlabtw/hicmaptools
Juicer and Juicebox	Durand and Robinson et al.^115,116^	https://github.com/aidenlab/juicer https://aidenlab.org/juicebox/
IGV	Robinson et al.^117^	https://igv.org/
Kallisto v0.45.0	Bray et al.^118^	https://pachterlab.github.io/kallisto/
MultiQC	Ewels et al.^119^	https://multiqc.info/
HiCExplorer	Ramirez et al.^60^	https://hicexplorer.readthedocs.io/en/latest/
pyGenomeTracks	Lopez-Delisle et al.^120^	https://pygenometracks.readthedocs.io/en/latest/
clusterProfiler	Yu et al.^121^	https://bioconductor.org/packages/release/bioc/html/clusterProfiler.html
ComplexHeatmap	Gu^122^	https://jokergoo.github.io/ComplexHeatmap-reference/book/
circlize	Gu^123^	https://jokergoo.github.io/circlize_book/book/
ggplot2	Wickham et al.^124^	https://ggplot2.tidyverse.org/
FAN-C	Kruse et al.^125^	https://fan-c.readthedocs.io/en/latest/getting_started.html#installation
Coolpup.py	Flyamer et al.^126^	https://github.com/open2c/coolpuppy
ggalluvial	Brunson et al.^127^	https://corybrunson.github.io/ggalluvial/index.html
Adobe lllustrator/Photoshop 2022	Adobe	https://www.adobe.com/
matplotlib	Hunter et al.^128^	https://matplotlib.org/stable/index.html
ImageJ v2.3.0	NIH	https://ImageJ.net/
Seurat v4.3	Hao et al.^129^	https://satijalab.org/seurat/

## Highlights

- Major change in 3D genome correlates with emergence of late-born retinal cell types
- TAD boundaries and inter/intra-TAD interactions are dynamic and stage specific
- Enhanced chromatin looping forms growing networks with retinal marker and TF genes
- Genome topology of organoids progressively resembles that of the adult human retina

## INCLUSION AND DIVERSITY STATEMENT

We support inclusive, diverse, and equitable conduct of research.
