# Supplementary material for: Stage-specific dynamic reorganization of genome topology shapes transcriptional neighborhoods in developing human retinal organoids
Source: Cell Rep. Author manuscript; Available in PMC 2024 Jan 16. (PMC10790351; doi:10.1016/j.celrep.2023.113543)
Supplement: 1 [file NIHMS1954792-supplement-1.pdf]

Cell Reports, Volume *42*

Supplemental information

Stage-specific dynamic reorganization of genome topology shapes transcriptional neighborhoods in developing human retinal organoids

Zepeng Qu, Zachary Batz, Nivedita Singh, Claire Marchal, and Anand Swaroop

SUPPLEMENTARY FIGURES

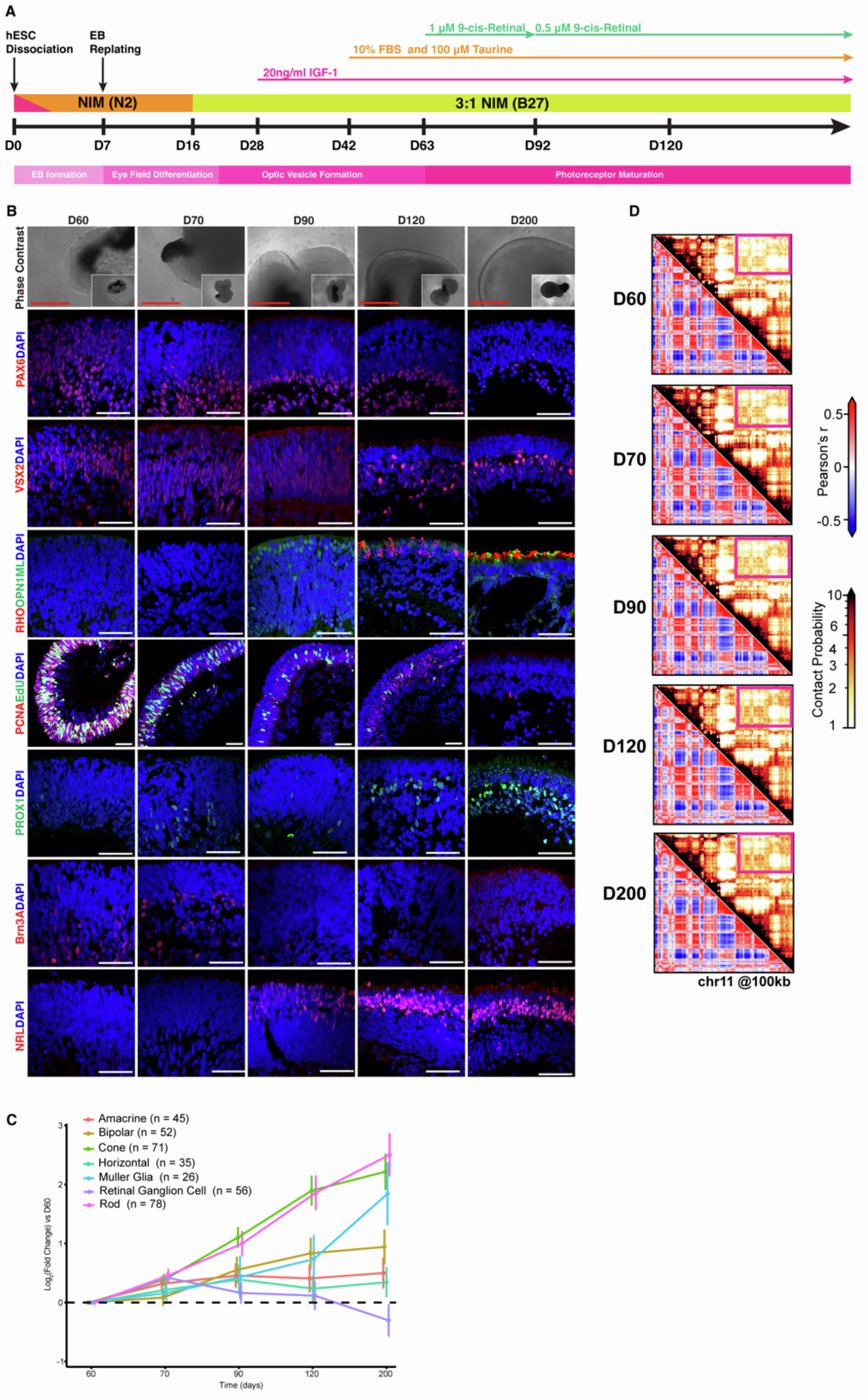

Figure S1. Differentiation and characterization of retinal organoids derived from human pluripotent stem cells, related to Figure 1.

(A) Schematic illustrating the protocol used to generate retinal organoids (see STAR Methods for additional details). EB, embryoid body; hESC, human embryonic stem cell; NIM, neural induction medium.

(B) Morphology of developing retinal organoids (first row) and representative immunofluorescence images of lineage markers in retinal organoids during differentiation. Scale bars: red, 1000 µm; white, 50 µm.

(C) Expression changes for retinal cell type marker genes relative to D60. Data are represented as mean ± SE. The number of genes counted for each cell type is shown.

(D) Hi-C contact maps of chromosome 11 at 100 kb-resolution for each time point: Knight-Ruiz (KR) balanced contact probability matrices (upper right triangle) and matching Pearson's correlation matrices (lower left triangle). The pink square represents a region of interest with increasing long-range contacts.

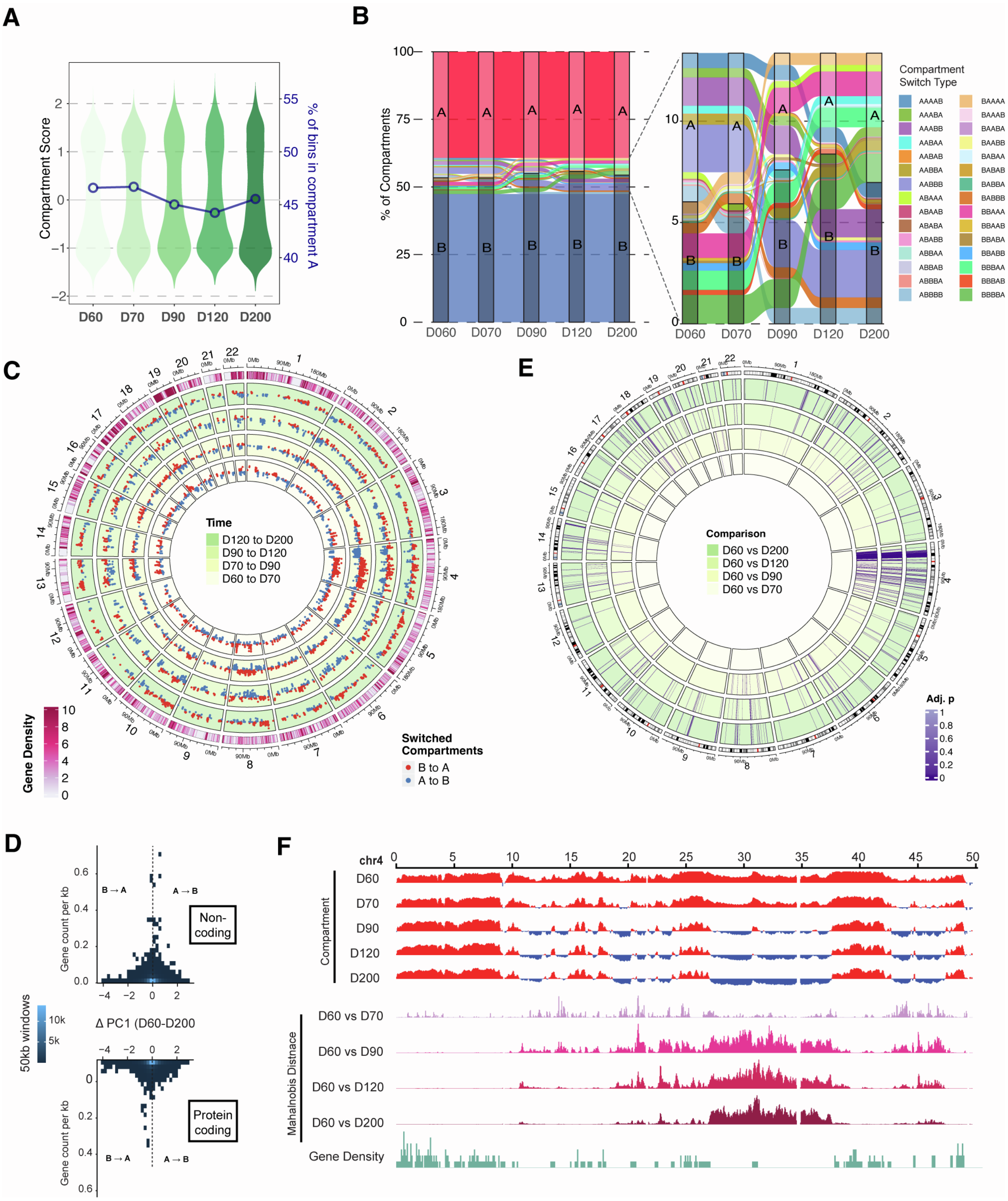

Figure S2. Compartment switches are widespread throughout retinal organoid development, related to Figure 2.

(A) Distribution of compartment scores (violin plots, left axis) and total percent of 50kb bins in A compartment by time point (line plot, right axis).

(B) Sankey plot of compartment status across development. Inset shows all compartment switch types.

(C) Outermost ring shows gene density across autosomes. Inner four rings mark the locations of compartment switches at each time point relative to immediately preceding time point.

(D) Density plot showing change in compartment score between D60 and D200 for each 50kb window across the genome grouped by non-coding (upper) and protein-coding (lower) gene density. Color represents the number of 50kb windows at a given combination of compartment change and gene density.

(E) Each ring shows the adjusted *p* value associated with compartment shifts in autosomes as calculated by dcHiC.

(F) Region on chromosome 4 (Chr4:0-50Mb) with high concentration of compartment switching. Upper five tracks show compartment score. Middle four tracks show Mahalanobis distance as calculated by dcHiC. Bottom track shows gene density by 50 kb window.

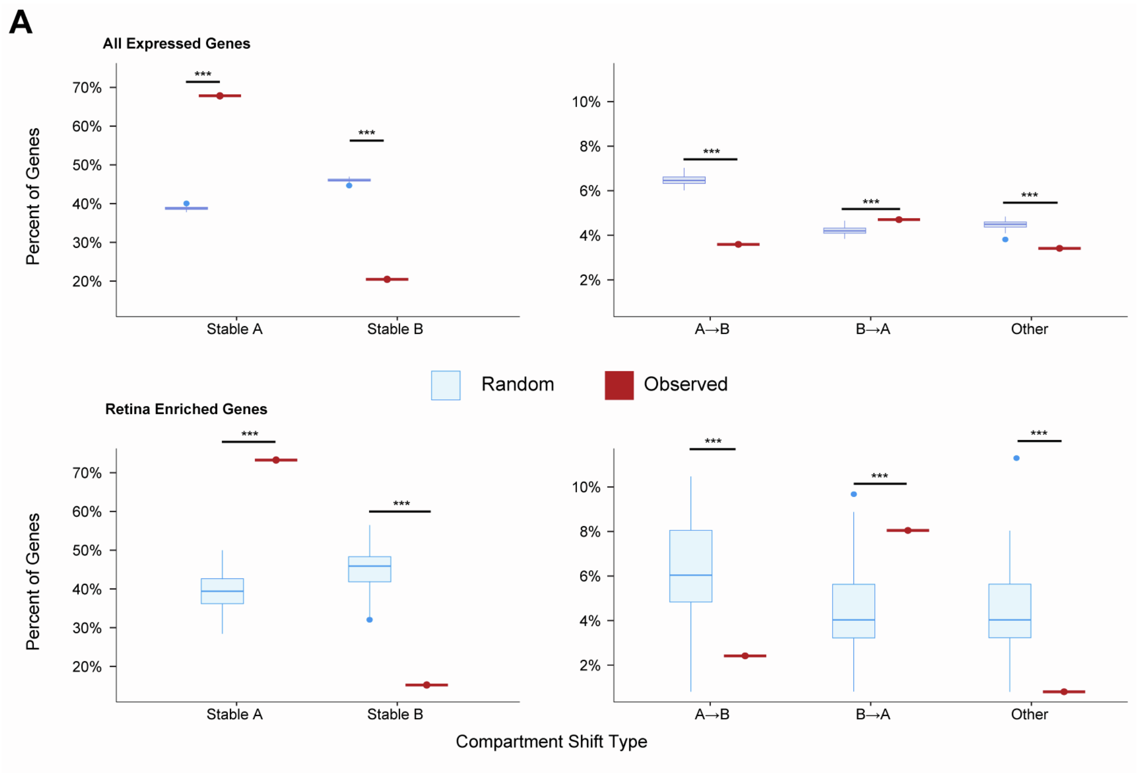

Figure S3. Expressed and retina-enriched genes are concentrated in A compartments, related to Figure 3.

(A) Proportion of all expressed (top) and retinal-enriched (bottom) genes in each compartment type. Red bars represent real values. Blue bars represent distribution of values obtained from 100 sets of shuffled compartment scores. *** $p < 0.01$ with one-sided t-test.

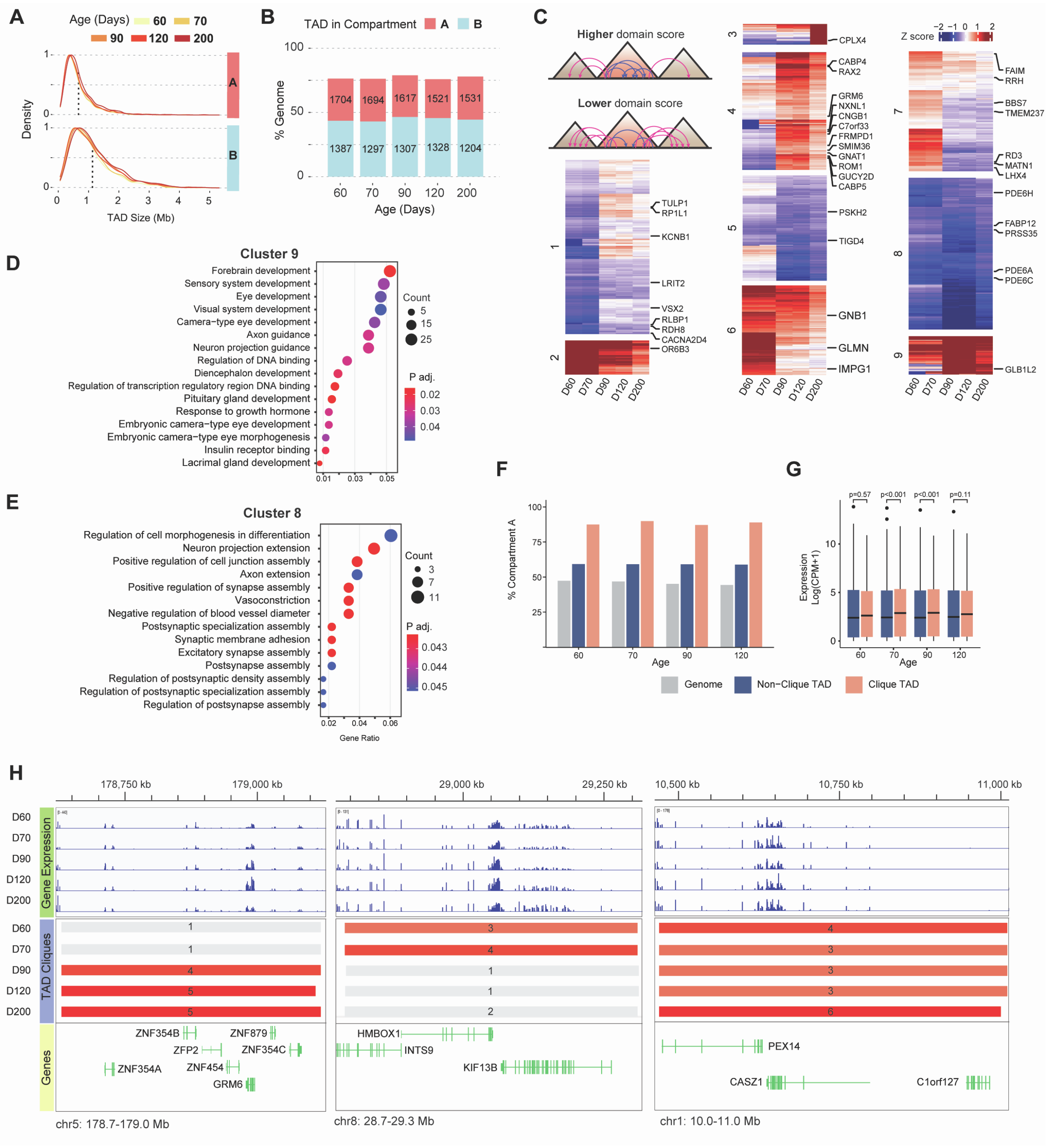

Figure S4 Dynamic changes of TAD structure and its regulation on gene expression during retinal organoid, related to Figure 4.

(A) Distribution of TAD sizes throughout all time points in A (top) and B (bottom) compartments. The grey dashed lines show the mean across all samples.

(B) Proportion of the genome within A and B compartment TADs throughout development. Value on bars indicate the number of TADs.

(C) Schematic illustrating domain score calculation (top left). Arrows indicate intra-TAD (blue) or inter-TAD (red) interactions. An increased domain score indicates that a TAD has increased intra-TAD contacts and relative to inter-TAD contacts. Clustered heatmap shows domain scores for stable TADs observed across all five time points with the location of genes of interest marked alongside.

(D and E) GO term enrichment for cluster 8 (consistently low domain score) and Cluster 9 (consistently high domain score).

(F) Proportion of genome, TADs outside cliques, and TADs in cliques which overlap with A compartment by time point.

(G) Expression of genes overlapping TADs in cliques and outside cliques. Boxplots represent the median and interquartile range (IQR); whiskers mark 1.5x the IQR; data beyond 1.5x the IQR are plotted as individual points. Significance evaluated by Kolmogorov-Smirnov test.

(H) Select regions with TAD cliques. The five top tracks show gene expression. The five lower tracks show a stable TAD with a value indicating the number of TADs making significant contact at a given time point. TADs interacting with >3 TADs are identified as TAD cliques (red). The examples illustrate different TAD cliques dynamics: formation of a TAD clique over *GRM6*, the gene initiating rod bipolar cell signal transduction (left), loss of a TAD clique over *KIF13B*, a kinesin protein regulating axon formation in hippocampal neurons (middle), maintained TAD clique over *CASZ1*, a zinc finger transcription factor

required for establishing the inverted chromatin organization within rod photoreceptors (right).

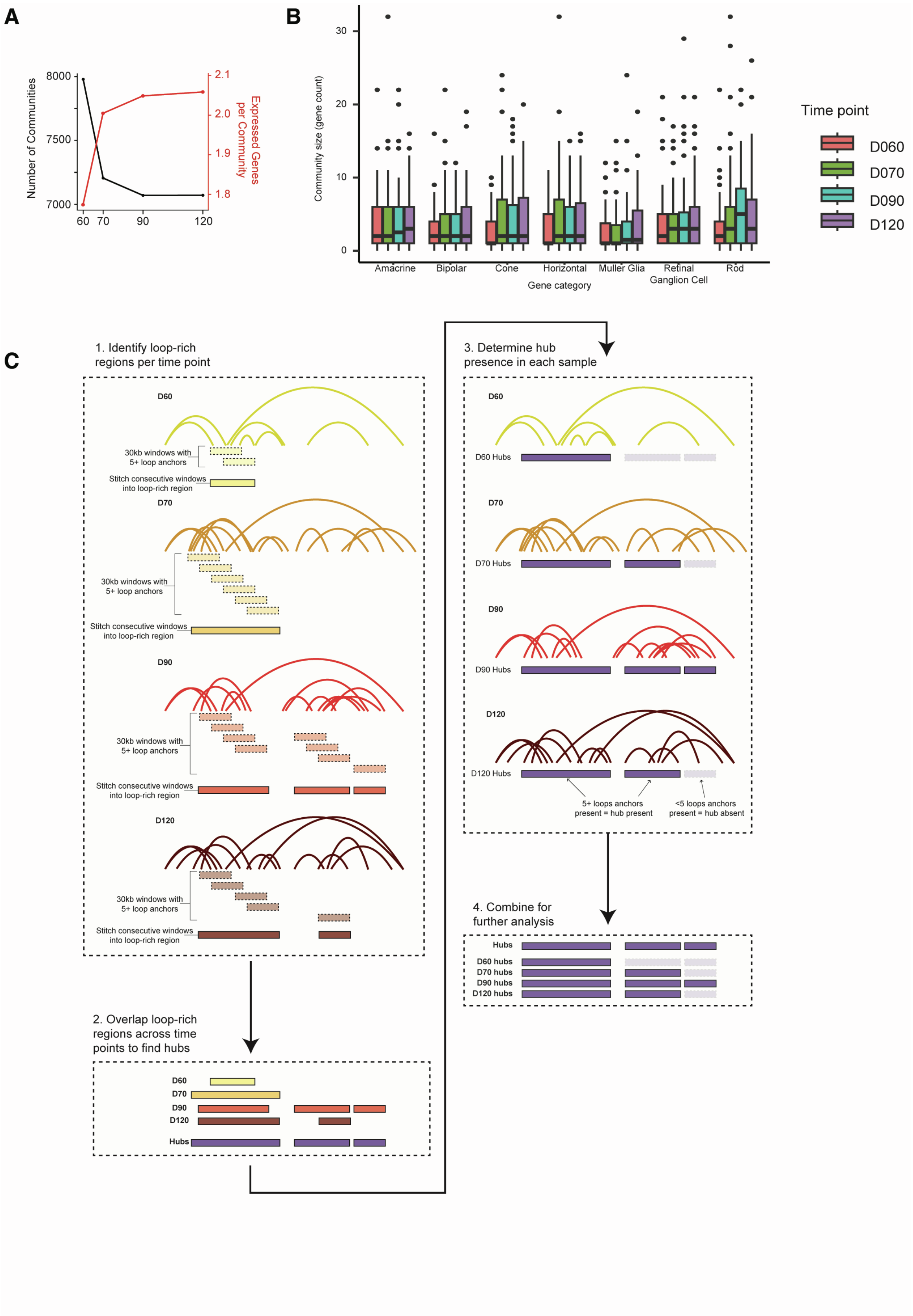
A
Number of Communities
Expressed Genes per Community
B
Community size (gene count)
Amacrine
Bipolar
Cone
Horizontal
Muller Glia
Retinal Ganglion Cell
Rod
Gene category
Time point
D060
D070
D090
D120
C
1. Identify loop-rich regions per time point
D60
30kb windows with 5+ loop anchors
Stitch consecutive windows into loop-rich region
D70
D90
D120
2. Overlap loop-rich regions across time points to find hubs
Hubs
3. Determine hub presence in each sample
D60 Hubs
D70 Hubs
D90 Hubs
D120 Hubs
5+ loops anchors present = hub present
<5 loops anchors present = hub absent
4. Combine for further analysis
D60 hubs
D70 hubs
D90 hubs
D120 hubs

Figure S5 Gene expression dynamics at chromatin hubs during retinal organoid differentiation, related to Figure 5.

(A) Total number of gene communities detected by time point (left axis, black) and the mean number of expressed genes contained within each community (right axis, red).

(B) Size distribution for gene communities containing retinal cell type marker genes. Boxplots represent the median and interquartile range (IQR); whiskers mark 1.5x the IQR; data beyond 1.5x the IQR are plotted as individual points.

(C) A schematic diagram describes the definition of chromatin hub (see STAR Methods for additional details).
